# 2D Nanomaterials for Effective Energy Scavenging

**DOI:** 10.1007/s40820-021-00603-9

**Published:** 2021-03-03

**Authors:** Md Al Mahadi Hasan, Yuanhao Wang, Chris R. Bowen, Ya Yang

**Affiliations:** 1 CAS Center for Excellence in Nanoscience, Beijing Key Laboratory of Micro-Nano Energy and Sensor, Beijing Institute of Nanoenergy and Nanosystems, Chinese Academy of Sciences, Beijing, 100083 People’s Republic of China; 2 SUSTech Engineering Innovation Center, School of Environmental Science and Engineering, Southern University of Science and Technology, Shenzhen, Guangdong 518055 People’s Republic of China; 3 Department of Mechanical Engineering, University of Bath, Bath, BA27AK UK; 4 School of Nanoscience and Technology, University of Chinese Academy of Sciences, Beijing, 100049 People’s Republic of China; 5 Center on Nanoenergy Research, School of Physical Science and Technology, Guangxi University, Nanning, 530004 People’s Republic of China

**Keywords:** 2D nanomaterials, Solar energy, Tribo-/piezo-/thermo-/pyro-electricity, Osmotic power generation, Self-powered sensor

## Abstract

The development of a nation is deeply related to its energy consumption. 2D nanomaterials have become a spotlight for energy harvesting applications from the small-scale of low-power electronics to a large-scale for industry-level applications, such as self-powered sensor devices, environmental monitoring, and large-scale power generation. Scientists from around the world are working to utilize their engrossing properties to overcome the challenges in material selection and fabrication technologies for compact energy scavenging devices to replace batteries and traditional power sources. In this review, the variety of techniques for scavenging energies from sustainable sources such as solar, air, waste heat, and surrounding mechanical forces are discussed that exploit the fascinating properties of 2D nanomaterials. In addition, practical applications of these fabricated power generating devices and their performance as an alternative to conventional power supplies are discussed with the future pertinence to solve the energy problems in various fields and applications.

## Highlights

- An introduction to the range of 2D nanomaterials and their advantages for energy scavenging applications.
- A variety of methods are presented for converting solar, mechanical, thermal, and chemical energies into electrical energy.
- A discussion of exclusive applications that exploit 2D nanomaterials for self-powered sensor devices.

## Graphical abstract

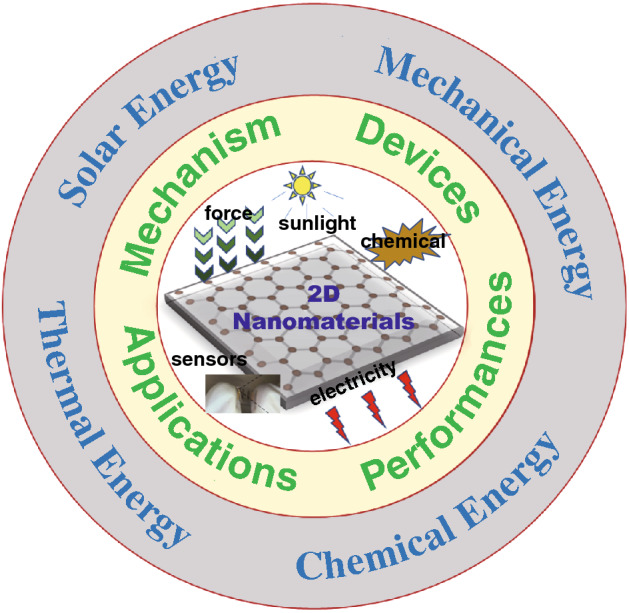

## Introduction

Energy scavenging continues to develop in every sector as a means to supply power for applications ranging from the home to industry. The demand for energy resources is increasing rapidly as scientists continue to develop alternatives to batteries for low-power electronics and sensors to larger-scale power in an attempt to ensure a stable energy supply in the future. A unique door has opened up for scientists with the discovery of graphene through exfoliating graphite [1], and the investigation of 2D nanomaterials has become a developing field. Researchers have been working to explore the future demand for energy in an effective and environment-friendly way. 2D nanomaterials are referred to as the materials having an ultra-thin layered crystalline structure with covalent bonding in the intra-layer and Van der Waals bonding in the inter-layer [2]. The large surface area-to-volume ratio and its atomically thin nature lead to 2D nanomaterials exhibiting dramatically different behaviors to its bulk state with novel characteristics due to quantum confinement [3].

In ultra-thin nanomaterials, such as graphene, with a defect-free crystal structure, its electrons need to pass a shorter path; this leads to a very high charge carrier mobility and an ultra-high electrical conductivity [4]. These properties make them an attractive option in fabricating a range of exciting nanoelectronic devices. The ultra-high transverse area and ultra-thin structure of two-dimensional nanomaterials provide the maximum amount of surface atoms, which creates an improved environment for specific applications such as photocatalysis, photovoltaics, and supercapacitors, where an ultra-high surface area represents a significant performance parameter for device effectiveness. As a result of these novel and unusual properties, 2D nanomaterials are being investigated for energy conversion and storage [5] with excellent performance and potential. Scientists are now moving to exploit these intriguing nanomaterials for real-life applications. For energy scavenging applications, 2D nanomaterials are being used in (i) solar energy scavenging such as photovoltaic cells [6], perovskites [7], photocatalysis [8]; (ii) mechanical energy scavenging such as triboelectric [9] and piezoelectric devices [10]; (iii) thermal energy scavenging such as thermoelectric [11] and pyroelectric systems [12]; and (iv) chemical energy scavenging such as osmotic power generation [13]. 2D nanomaterial-based nanogenerators are potentially an attractive option for large-scale power generation from sustainable sources such as wind power, ocean waves, and rolling wheels [14]. In addition, the generated power from these nanogenerators can supply power for the operation of portable electronic devices [15] that can facilitate multi-functionally in real-life applications such as body motion sensing and code transmission by a single generator which scavenges energy from the human body [16].

A number of excellent reviews have been published on 2D nanomaterials based on device fabrication for power conversion, storage technologies, and sustainable energy applications [17–19]. However, these existing reviews are limited to specific materials, mechanisms, and application areas. In this review, the area of 2D nanomaterials for energy scavenging devices for all the available energy resources is summarized with their performance analysis to provide a broad insight into this rapidly developing area. In addition, the devices that are being fabricated using 2D nanomaterials for self-powered devices are discussed. This includes a range of sensors that aim to exploit 2D nanomaterials. In the first section of this review, an overview of current nanomaterials is discussed with their unique and essential properties, which are important for various energy scavenging techniques and suitable fabrication processes. In the second section, the range of energy scavenging mechanisms and device performance parameters will be explained in detail with their structure and effectiveness in replacing conventional energy sources. In addition, self-charging supercapacitors will be summarized based on 2D nanomaterials as a storage substitute. The third section describes specific real-life applications where 2D nanomaterial-based devices are being used as an alternative source of power for their operation in sensors both for industrial, health, and environmental monitoring purposes. The conclusions will provide insights into future directions for these new materials in energy scavenging research.

## 2D Nanomaterials for Energy Scavenging Devices

**Fig. 1:**
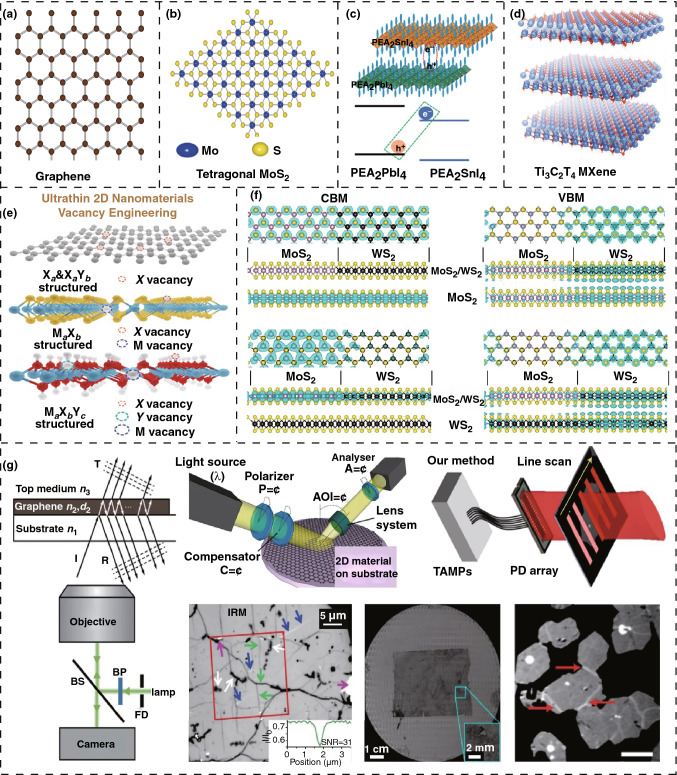
Introduction of some nanomaterials and some modifications. **a** A single-layer graphene sheet. **b** Transition metal dichalcogenide. **c** Heterostructure of 2D perovskite. **d** Ti_3_C_2_T_x_ MXene. **e** Diagram of 2D nanomaterial structure and type of vacancy(s). **f** CBM and VBM’s charge density in lateral heterostructure of MoS_2_/WS_2_. **g** Characterization techniques to detect abnormalities using optical methods. Figures reprinted with permission from: **a** Ref. [23], © 2014 American Institute of Physics; **b** Ref. [30] © 2016 Elsevier; **c** Ref. [37], © 2020 Nature; **d** Ref. [38], © 2019 Wiley; **e** Ref. [54], © 2020 Wiley; **f** Ref. [56], © 2015 American Chemical Society; **g** Ref. [28], © 2019 Nature

Among the range of 2D nanomaterials, graphene is the most investigated material for energy scavenging and device fabrication due to its excellent charge carrier mobility and low-cost production [20]. It is atomically thin with *sp*^2^ hybridization of its carbon atoms; Fig. 1a shows an image of a single-layer graphene sheet. Due to this configuration, graphene is highly transparent that can be an alternative to the transparent conductive oxide (TCO) in organic solar cells between the glass surface and organic materials [21]. It can also enhance electron transport and the dissociation of excitons between the heterojunctions of solar cells. A smooth and planar surface provides a low contact resistance that can reduce the potential drop and prevent leakage currents at the interfaces of p-type and n-type material solar cells [22]. Due to the high scattering of phonons in graphene, the thermal conductivity remains very high at room temperature, which is an important requirement for effective thermal energy scavenging [23]. The heterostructure of graphene enables it to metalize the 1D edges of the 2D layer graphene. Its surface geometry allows the complete separation of contacts, which leads to high-performance electronic devices [24]. The outstanding conductance in terms of electrical and thermal properties, with an ultra-wide surface area, leads graphene to be a material of interest for dye-sensitized solar and fuel cells [25]. Graphene-related materials, such as graphene oxide (GO), have a very high Young’s modulus and an excellent dielectric constant, enabling it to be a good option for the piezoelectric energy conversion process to utilize mechanical energies [26]. For a greater electronic device output, graphene-enabled or directed nanomaterials have been investigated for large-scale device integration to understand nanoelectronic industry-scale applications [27]. Considering the low cost, high lifetime, and modification capability of the properties, the future of graphene is mesmerizing both in terms of energy scavenging and in terms of storage technologies [25]. To meet commercial demands from a wide variety of patents and applications, graphene is to be produced at a scale of thousands of tons every year [28].

2D transition metal dichalcogenides (TMDs) nanomaterials are similar to graphene. These nanomaterials have been investigated broadly in energy scavenging and sensor applications. Metal dichalcogenides (MX_s_): where M denotes the transitional metals and X_s_ represents S, Se, or Te, and it can be in both mono-layer and multilayer form [29]. During the exfoliation of multilayers, due to the interaction of *s-P*_*z*_ orbitals, the bandgaps of single-layer become wider, which enables a transformation of indirect bandgap into direct bandgap, thereby providing excellent photoluminescence. The wide bandgap is an important property to avoid current leakage in piezoelectric materials and photoluminescence provides a better solar energy harvesting mechanism [29]. A diagram of the single-layer crystal structure of tetragonal MoS_2_ is shown in Fig. 1b. The ultrathin two-dimensional nanomaterials based on MoS_2_ show highly favorable properties, such as an excellent density of states of electrons and holes with a very high Seebeck coefficient [30]. According to its electrical properties, its members are *insulators* such as HfS_2_, *semiconductors* such as MoS_2_, *semi-metals* such as WTe_2_, and *true metal* such as NbS_2_, thereby covering a great diversity of the electrical spectrum in response to the oxidation level along with the adjustments of transition metal atom [31]. TMD-based devices are also capable of high-frequency operations due to their significant bandgap, which is important for communication and data processing applications [32]. Two-dimensional TMDs offer many tunable properties functionalization with various polymers, thereby making them excellent candidates for flexible and transparent electronics applications with improved mechanical efficiency [33].

2D perovskite nanomaterials have elevated light-harvesting properties. Due to these properties, these materials are being extensively and increasingly investigated in solar energy scavenging applications. For stabilizing perovskites, both formamidinium (FA)- and phenylformamidinium (PFA)-originated cations are working as a substitute of surface patches. While formamidinium cations are better due to their attenuated band gaps and stability, the mixed cation 2D perovskites show excellent performance both in terms of device improvement and in terms of thermal stability. This performance is achieved as a result of the enhancement of surface flaws and avoiding the rearrangement of the hole transfer layer and perovskites layer at adjacent surface edges [34]. The nonlinear scattering and photocarrier recombination are comparatively reduced in ultrathin 2D perovskites, which is favorable for the investigation of intrinsic optical properties [35]. The triangle and hexagonal shapes of the high-quality 2D perovskite crystals facilitate tunable photoluminescence enabling wider applications in optoelectronics [36]. Charge-transfer excitons (CTEs) can be generated from the heterostructure of 2D perovskites, as shown in Fig. 1c, using a combination of PEA_2_PbI_4_: PEA_2_SnI_4_ which operates as a host and guest, respectively. This process leads to tuning of the properties of the perovskite that can provide opportunities to develop advanced optoelectronic devices [37].

Recently, 2D MXene nanomaterials are receiving significant interest as a result of the strength of their fascinating electrical, plasmonic, optical, and thermoelectric characteristics. These properties, in addition to their extended effective surface, have made them attractive materials for electronic, electrochemical, and catalytic applications for energy scavenging. MXenes are a group of 2D nanomaterials that consist of transition metals carbides, nitrides, and carbonitrides. Figure 1d shows Ti_3_C_2_T_x_ MXene synthesized from a MAX phase, in which M refers to a transition metal, A symbolizes the main element cluster, and X refers to carbon (C) or nitrogen (N) [38]. 2D thin films synthesized from the deposition of MXene dispersion in a layer-by-layer way show high optical transmittance (more than 80%), which is an important requirement for photoactive materials for the fabrication of displays and PV (photovoltaic) cells [39]. Due to the conductivity of carbide core and accumulation of dipoles on the surface of MXene fillers and polymer matrix, MXenes exhibit good electrical conductivity and dielectric properties under the applied electric field [40]. The outstanding surface chemistry makes MXenes and its nanocomposites an active field of study for electrochemical energy scavenging, such as electrolytic water splitting [41]. In addition, the increased surface charge density is of interest for extending the nanofluidic energy conversion such as osmotic power generation by playing an important role in modulation of the ion diffusion procedure between two liquids having a different level of salinity [13]. Existing published research from the web of science databases shows that MXenes have already attracted significant attention from the scientific community for energy scavenging technologies.

2D metal–organic frameworks (MOFs) have a structure of crystal-like materials, where the metallic node is bound with a variety of organic ligands. Due to their porous structure and surface chemistry, MOFs have excellent performance in electrolytic techniques such as the hydrogen evolution reaction (HER), oxygen evolution reaction (OER), carbon dioxide reduction reaction (CRR), oxygen reduction reaction (ORR), and urea oxidation reaction (UOR) [42]. The functionalization of MOFs with quantum dots (QDs) can extend the light-harvesting behavior, offering enhanced coverage of the light spectrum with adsorption of the visible region by QD-MOF hybridized fabricated devices [43]. Due to their tunable morphological structure, porous surface, and favorable electrochemical properties, 2D MOFs can be a convenient choice of material for energy scavenging, catalysis, and sensor applications with the opportunity of efficient device fabrication [44]. Due to the mixing of MOFs with a polymer matrix, its deposition on the support surface and applied mechanical compression exhibits limitations such as blockage of pores, destruction of the framework, or compound aggregation hampering device performance [45].

In addition to the mentioned 2D nanomaterials [21–45], many others are being investigated for energy scavenging, and the number continues to increase. 2D nanomaterials synthesized from metal oxide and layered hydroxide materials are favorable for energy scavenging applications. The materials include PV (photovoltaic) cells, photocatalysis, piezoelectric power generation, and fuel cells [46]. ZnO nanoleaves have been synthesized through simple chemical vapor deposition (CVD) that shows optimistic electromechanical properties for the application in NEMS (nanoelectromechanical system) devices [47]. Some metallic non-layered 2D nanomaterials, such as several pure metals and alloys and layered 2D nanomaterials, include germanene, silicene, stanene, antimonene, which are being investigated for solar cells, catalytic, surface plasmon resonance process for sensing and energy conversion applications [48]. By stacking 2D unilamellar nanosheets from bulk materials, some 2D superlattices are being investigated since they provide very attractive electrical properties for energy scavenging such as water-splitting and energy storage technologies [49]. Researchers are also considering a diverse range of innovative 2D nanomaterials such as bio-inspired nanomaterials from biological organs or components [50], 2D polymer nanomaterials [51], and rare-earth 2D nanomaterials from lanthanum (La) to lutetium (Lu) in the periodic table. In addition, researchers are considering composites [52] for energy scavenging and device fabrication.

With the enhanced diversity and advanced properties, 2D nanomaterials have unique characteristics such as functionalized hybridization [53], surface engineering [55], and many other techniques for tuning the specific characteristics according to the application requirements. To elevate the optoelectrical properties of two-dimensional nanomaterials, such as TMDs, techniques such as doping with ions and atoms, surface modification with organic molecules, surface adsorption with macromolecules, and surface hybridization with other nanostructures are being investigated for improved device performance [53]. In some instances, different types of vacancies, as shown in Fig. 1e, can be formed during the synthesis of the 2D nanomaterials under specific harsh conditions that can be turned into the localization of electrons, lattice distortion, electronic compensation, and disbanding and reconstructing chemical bonds. These types of adjustments provide unique physical and chemical properties, which opens new opportunities for innovative research in photocatalytic and electrolytic energy scavenging applications [54]. In the context of exploiting cation and anion vacancies, distortion, and disorder on 2D nanomaterials, the process of surface defect engineering can significantly increase the photocatalytic properties by enhancing both charge separation and light adsorption [55]. *Tunable carrier confinement* in 2D nanomaterials is a mechanism by which the application of mechanical strains leads to band alignment adjustment since a specific nanomaterial is always with an increasing valence band maximum (VBM) along with the degrading conduction band minimum (CBM). The intensity of charge for both VBM and CBM for a double-layered lateral heterostructure of MoS_2_/WS_2_ is presented in Fig. 1f, where a heterostructured MoS_2_/WS_2_ is at the head of the MoS_2_/WS_2_’s single-layered [56].

By implementing a hybrid nanostructure and using surface modification between the nanosheets of layered 2D nanomaterials, it is possible to tune the electrochemical properties, develop excellent electrical conductivity, and highly active surface chemistry for electrochemical energy scavenging applications and sensor fabrication [57]. Recently, *chirality* has also been introduced in 2D nanomaterials by liquid exfoliation with the appearance of the chiral ligands and investigation of its significance in optoelectronic and sensor technologies [58]. *Supermolecules* are also being investigated in 2D nanomaterials for extended performance and efficiency [59]. These tunable characteristics contribute to taking part in the principal role in the advancement of 2D nanomaterial-based energy scavenging applications and device fabrication research. For analyzing these characteristics, different types of characterization techniques are being applied, which are shown in Fig. 1g, where optical methods such as optical spectroscopy, interference reflection microscopy, scanning electron microscopy (SEM) are being used to locate voids, cracks, and determine the number of layers for exact characterization in a range of versatile applications [28]. As a result of their compact size, 2D nanomaterial-based nanogenerators can perform in a range adverse conditions in water and harsh environments [60].

## Mechanisms and Performance of 2D Nanomaterial-Based Energy Scavenging Devices

### Solar Energy

Future energy supplies should depend on the utilization of sustainable sources and solar energy aided with 2D nanomaterials, which has turned into a dynamic area of energy scavenging studies. The superconducting and exceptional electrical properties of 2D nanomaterials have made them an active area in the development of highly efficient photovoltaic (PV) cells [63, 64], perovskites solar cells [65, 66], polymer cells [67], photocatalysis, and water-splitting systems [72, 73]. The majority of 2D nanomaterials used with heterojunction-like graphene can be used on silicon surfaces, whose efficiency ranges from 15 to 17% [61]. The opportunity for tuning the properties of 2D nanomaterials has proven to be attractive in fabricating materials for solar energy harvesting devices. A comparative summary of the reported works based on 2D nanomaterials with the device structure, fabrication methods, and outputs is given in Table 1.

**Table 1:** Comparative summary of reported work on solar energy harvesting including device structure, functional materials, fabrication techniques, and device outputs

Device structure	Functional materials	Fabrication techniques	Device outputs	References
	Graphene, Gallium nitride (GaN)	Metal–organic chemical vapor deposition (MOCVD)	V_oc_ = 0.225 V J_sc_ = 0.0257 mA cm^−2^ FF = 23% PCE = 0.0013%	[62]
	Graphene, Hexagonal boron nitride (h-BN)	Photolithography chemical vapor deposition (CVD)	V_oc_ = 0.547 V J_sc_ = 32.89 mA cm^−2^ FF = 54.2% PCE = 10.93%	[63]
	CdSeTe CdTe	MOCVD Vapor-transport deposition (VTD)	V_oc_ = 0.921 V J_sc_ = 30 mA cm^−2^ FF = 80% PCE = 20.8%	[64]
	Phenylethylammonium lead iodide (PEA_2_PbI_4_)	Spin coating	V_oc_ = 1.13 V J_sc_ = 24 mA cm^−2^ PCE = 20.64%	[66]
	Graphene WO_x_ WSe_2_ MoS_2_	Beam lithography E-beam deposition	V_oc_ = 0.48 V I_sc_ = 0.69 µA FF = 45% PCE = 5%	[67]
	rGO/PANI-Ru hybrid nanocomposite	Spin coating	V_oc_ = 0.732 V J_sc_ = 14.59 mA cm^−2^ FF = 63.6% PCE = 6.8%	[68]

**Fig. 2:**
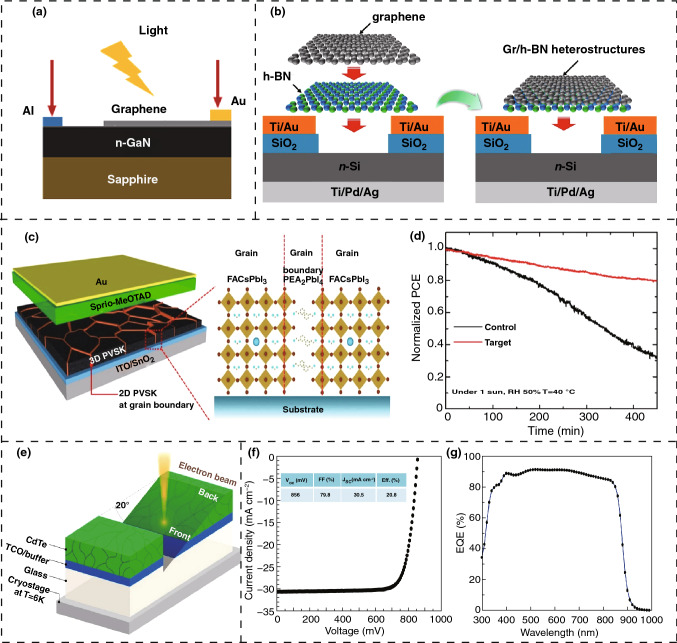
Mechanism of solar energy scavenging through solar cells. **a** Diagram of nanodevice based on heterojunction of Graphene/n-GaN. **b** Schematic of solar cell fabricated through one- and two-step method. **c** Two-dimensional perovskite at grain boundary of device **d** Tracking of highest powerpoint of the device with no encapsulation under normal condition. **e** Sample excitation with electron beam. **f** Current density–voltage curve for the device with As doping without any Cu addition. **g** Relative EQE values with wavelengths. Figures reprinted with permission from: **a** Ref. [62], © 2017 American Institute of Physics; **b** Ref. [63], © 2016 Elsevier; **c**, **d** Ref. [66], © 2018 Nature; **e**, **f**, **g** Ref. [64], © 2019 Nature

Figure 2a shows a schematic of a typical fabrication of a photovoltaic device fabricated with graphene/n-GaN on a sapphire substrate with gold (Au) and aluminum (Al) contacts having a calculated open-circuit electric potential of 0.224 V. It has an efficiency of 0.0013% along with a fill factor (FF) of 23% [62]. The fabrication stage may include additional processing steps to introduce new surface or interface engineering and obtain greater efficiencies and improved performances. In Fig. 2b, an interface modification is introduced by the addition of a hexagonal boron nitride (h-BN) layer. Thus, a larger electrical conductivity of h-BN decreases the series resistance of the solar cell, thereby resulting in a higher open-circuit voltage [63]. Doping with different cations or anions such as arsenic (As) in a cadmium telluride (CdTe) solar cell is another strategy to achieve high-efficiency solar cells. Figure 2e represents the process of analyzing the cathode luminescence by applying an electron beam to find the undoped region with the level of excitonic emission [64]. The overall efficiency of the solar cell doped with As, without any anti-reflection coating, is 20.8%, which is shown in Fig. 2f along with the current density–voltage characteristics, and Fig. 2g delineates the related external quantum efficiency (EQE) [64].

**Fig. 3:**
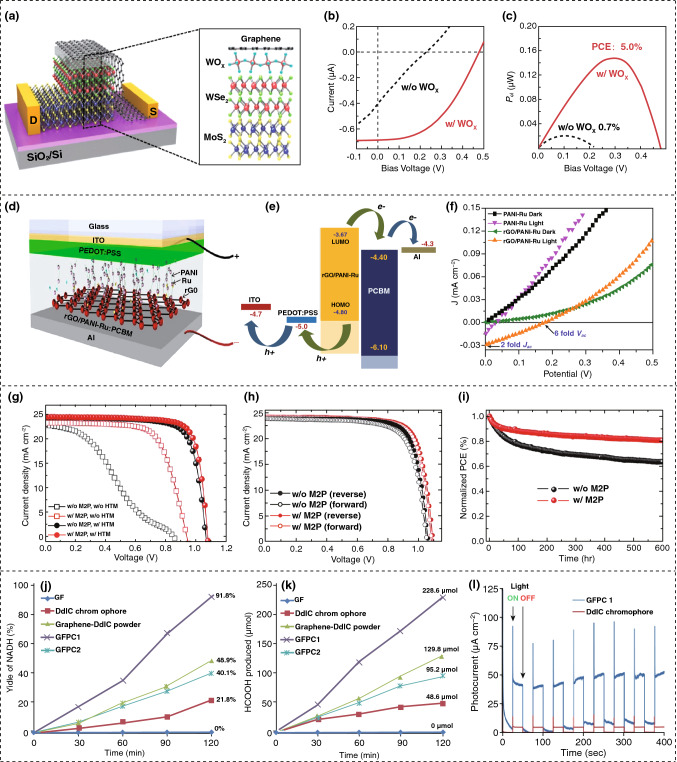
Solar energy harvesting mechanism and performances of the 2D nanomaterial-based scavenging devices. **a** Schematic of device with WOx layer. **b** Photovoltaic properties without (black) and with (red) WO_x_ being excited by laser ray. **c** Electrical power plot with extended photoconversion efficiency to 5%. **d** Schematic of the fabrication of perovskite solar cell having an electron donor of rGO/PANI-Ru, **e** PSC’s energy-generating curve. **f** Electrical characteristics of device. **g** Current density voltage characteristics under both M2P and OMeTAD condition. **h** Plot of current density and voltage with spiro-OMeTAD along with M2P condition. **i** Stable performance of device under inert surroundings. **j** NADH regeneration. **k** Formic acid generation from CO_2_. **l** Photocurrent characteristics by applying simulated 1 sun illumination. Figures reprinted with permission from: **a**, **b**, **c** Ref. [67], © 2020 American Chemical Society; **d**, **e**, **f** Ref. [68], © 2017 Nature; **g**, **h**, **i** Ref. [69], © 2020 Wiley; **j**, **k**, **l** Ref. [77], © 2018 Nature. (Color figure online)

2D perovskites materials are becoming the epicenter of solar energy scavenging research as they are more favorable for many aspects, for instance the primary light-absorbing agent, passivation layer, cover coating, an anti-reflection coating, and more [65]. 2D nanomaterials have less volatility and have more hydrophobic organic ions than 3D materials, which leads to greater stability in thermal and chemical environments. Figure 2c provides a schematic of a solar device, where 2D formamidinium lead iodide (FAPbI_3_) is formed on the grain boundary of 3D perovskite. This structure provides better stability, as shown in Fig. 2d, which describes the photon conversion efficiency (PCE) measured by an encapsulated control and target device under continuous light [66]. The examination of Fig. 2d indicates that the target device shows less degradation of the maximum power point, which is only 20% rather than 68% for the case of the control device for 450 min. Thus, the efficiency of the perovskites has been extended to 19.77%. Figure 3a represents a photovoltaic device in which an atomically thin WOx layer is applied between the WSe_2_-MoS_2_ heterojunction to increase the photo-responsivity of the device [67]. The device characteristics are illustrated in Fig. 3b, wherein the output voltage, *V*_oc_ (open-circuit voltage), is elevated to 0.48 V from 0.23 V. More importantly, the overall power conversion efficiency is enhanced dramatically to 5% from only 0.7%, as shown in Fig. 3c [67].

A polymer solar cell based on pyridyl benzimidazole incorporated with Ru complex imprinted polyaniline assembly (PANI-Ru) is investigated using inoculating reduced graphene oxide (rGO). The device performance increases significantly because of the excellent electrical properties of rGO [68]. The designed polymer solar cell is shown in Fig. 3d, where the rGO/PANI-Ru: PCBM works as a photosensitive coating. The energy levels in the device are indicated in Fig. 3e, and the resulting value of HOMO and LUMO is − 4.8 and − 3.67 eV, respectively. In addition, Fig. 3f displays the current density–voltage (*J*–*V*) properties, where it is observed that there is no current density (*J*_sc_) in the device under dark conditions [68]. The application of multi-functional 2D perovskites (M2P) with the hole transporting material (HTM) along with 2,2′,7,7′-tetrakis-(*N*,*N*-di-p-methoxyphenyl-amine)-9,9′-spirobifluorene (spiro-OMeTAD) light absorber increases both the effectiveness and the stability since M2P triggers faster hole transport and provides a passivation effect due to its randomly oriented crystal structure [69]. It is clear from Fig. 3g, h that the fabricated device is dominating in terms of performance for all the criteria such as open-circuit voltage (*V*_oc_), current density (*J*_sc_), fill factor (FF), and photoconversion efficiency (PCE). This happens when it undergoes M2P deposition at both stages of perovskite solar cells in accordance with M2P and spiro-OMeTAD conditions. In addition, in the stage of spiro-OMeTAD on the basis of M2P requirements under the reverse and forward scan of the device, the stability of the perovskite solar cell improves by maintaining a maximum power point tracking to 80%, compared to 64%, in the case of applying M2P which is shown in Fig. 3i [69]. The highest efficiency from a perovskite solar cell to date is 25.2% [70]. Researchers are applying new materials and technologies such as applying additive engineering during film formation, introducing modifiers to elevate electron–hole transport, and changing the surface chemistry for a more efficient and stable perovskite solar cell [71].

In addition to PVs and PSCs, water splitting is a major concern for harvesting solar energy using 2D nanomaterials, in particular materials such as 2D phosphorene [72], 2D polymers [73], 2D porous TiO_2_ single-crystalline materials [74], and other 2D layered materials [75]. Water splitting uses sunlight and water to produce hydrogen fuel with specific surface redox reactions that are aided by the bandgap energy level of the nanomaterial [75]. The water-splitting process includes both the photoelectrochemical (PEC) and photocatalytic reaction (PC), but PEC is more attractive due to the production of O_2_ and H_2_ separately at different electrodes, thereby reducing the problem of oxygen and hydrogen separation [76]. A graphene-based hybrid artificial photosynthesis has been introduced by integrating a photocatalyst and biocatalyst for producing fuel from CO_2_ with a 225% increase in efficiency compared to a conventional graphene film-based photocatalyst [77]. Figure 3j, k shows the photocatalytic activity and production of NADH (nicotinamide adenine dinucleotide (NAD) + hydrogen (H)) and formic acid from CO_2_, which is derived from GF, DdIC chromophore, granulated graphene-DdIC, GFPC 1 (graphene film photocatalyst 1), and GFPC 2 (graphene film photocatalyst 2). It is clear from the graph that the graphene film photocatalyst is more efficient. The photocurrent evaluation in Fig. 3l indicates that GFPC has 10 times more current than DdIC chromophore, suggesting that GFPC is more effective at photocatalytic energy scavenging [77]. Harvesting solar energy effectively, 2D nanomaterials face difficulties in fabricating high-quality PSCs related to long-term and operational stability [66]. In addition, low-cost mass production, low efficiency, and poor light-harvesting properties remain a challenge for scientists. The excellent electrical properties, with no toxicity, are leading 2D nanomaterials to improve the efficiency of 2D nanomaterials for solar energy harvesting as a clean energy solution.

### Triboelectric Power Generation

*Triboelectric* power generation is a process where a small amount of electrical charge is stored between two materials after some mutual mechanical movement. Although the amount of energy is small, it can play a significant role for mini- or micro-electronic devices by removing the need for a battery-powered supply, and it facilitates the creation of self-powered devices by scavenging sustainable energy from its surrounding environment. After the development of the triboelectric nanogenerator (TENG) to generate triboelectricity from surrounding mechanical energy by Wang et al. [78] and his group, the TENG has captured the interest of the scientific community to investigate the approach for energy harvesting applications [78]. The output power of the triboelectric nanogenerator depends on the charging behavior of the materials due to the arrangement of the relative polarity under an applied mechanical energy. The larger the relative distance according to their negative or positive polarity, the greater the charging properties, which has a significant role on device performance [79]. The output power of the triboelectric process depends on material thickness, surface irregularity, dielectric constant, the number density of states (DoS), and impurity content [79]. By providing an opportunity to achieve favorable electrical and mechanical properties, 2D nanomaterials are becoming promising in this field for the fabrication of TENGs for a range of possible applications and as a substitute for external power sources.

**Fig. 4:**
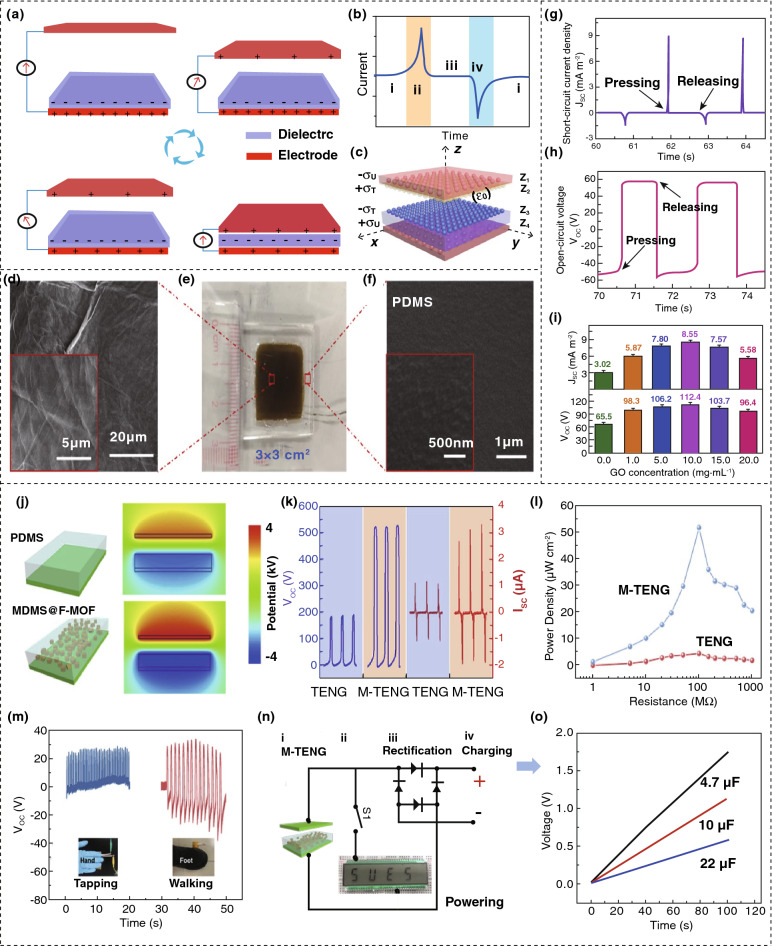
Mechanism of triboelectric power generation and device performances. **a** Basic operating principle of CS-TENG on applying an external force. **b** Generated current of device in a complete cycle. **c** 3D model of CS-TENG for analyzing output performances. **d** Schematic of GO by scanning electron microscope. **e** Image of GO LS-TENG. **f**, PDMS film imaged via SEM. **g** Output current when the circuit is shorted. **h** Output voltage when the circuit is open. **i** Concentration effect of GO on output current and voltage. **j** Distribution of electric fields generated by TENG and M-TENG using finite element simulation. **k** Electrical outputs from TENG and mTENG. **l** Output power according to load resistances. **m** Resulting voltage from human motion. **n** Required circuit diagram for supplying power to LCD and charging capacitors through rectification. **o** Powering curves for capacitors up to 100 s. Figures reprinted with permission from: **a**, **b**, **c** Ref. [80], © 2020 Elsevier; **d**, **e**, **f**, **g**, **h**, **i** Ref. [82], © 2019 Elsevier; **j**, **k**, **l**, **m**, **n**, **o** Ref. [83], © 2020 Elsevier

A typical diagram of the TENG configuration is represented by Fig. 4a, with its mechanism of operation, where the dielectric material polydimethylsiloxane (PDMS), mixed with TiO_2_ nanoparticles (NPs), is located between the upper and lower Al (aluminum) electrodes [80]. At the initial stage, the upper electrode is without a charge, but the lower electrode has a positive charge as the dielectric material is negatively charged following the triboelectric series [81]. When a perpendicular force acts upon the upper electrode, it touches the dielectric material, and positive charges flow through the circuit to the lower electrode, creating a fixed directional electrical pulse, as presented in Fig. 4b. The release of the external force results in an opposite inverse current signal, as expressed in Fig. 4b [80]. Figure 4c shows a three-dimensional (3D) mathematical formula for the estimation of device performance, where *ε*_0_ symbolizes air permittivity (free space), *ε*_2_ denotes the charge of that plane from the frictional effect of dielectric material, *σ*_T_ denotes the charge density of the virtual plane, *σ*_u_ indicates the charge density, and *z* symbolizes the electrical potential of electrodes [80]. The outputs of the TENG depend on the applied mechanical force and the weight ratio of TiO_2_ NPs. However, a large weight ratio of TiO_2_ can degrade the mechanical strength, resulting in reduced durability. However, an optimized device shows an effective response after 2000 cycles of operation and after four weeks.

However, a TENG with a liquid single electrode containing a 2D nanomaterial graphene oxide (GO) dispersion has been developed to provide improved stability, sensitivity, and flexibility [82]. Figure 4e shows an image of the designed device, where the GO dispersion plays the role of a liquid electrode. The morphology of GO is shown in Fig. 4d, with a lamellar 2D configuration along with a large number of folds, ripples, and high surface area, which leads to an elevated and effective charge transfer with a large degree of defects, as seen in Fig. 4f. The fabricated devices provide higher mechanical flexibility, improved deformability, and better mechanical properties. The output current density *J*_sc_ (short-circuit current) and voltage *V*_oc_ (open-circuit voltage) are presented in Fig. 4g, h, respectively. The effect of the level of GO dispersion concentration is also studied in the range from 1 to 20 mg mL^−1^; herein, the maximum output can be found at 10 mg mL^−1^ from Fig. 4i. The fabricated device is highly sensitive at frequencies as low as 0.45 Hz, producing a small, but detectable, current signal [82].

To further increase the efficiency of a TENG, many methods are being investigated. A fluorinated metal–organic framework (F-MOF) acting as a dual functional filler with a polydimethylsiloxane (PDMS) matrix to form PDMS@F-MOF is shown in Fig. 4j. The distribution of electric fields is studied, which increases the electrical output 11 times compared to conventional devices [83]. The output voltage *V*_oc_ (open-circuit voltage) along with current density *J*_sc_ (short-circuit current) in conventional PDMS (TENG) and PDMS@F-MOF (M-TENG) can be found in Fig. 4k; also, the power density is represented by Fig. 4l. It is also noticeable that the F-MOF mass ratio with PDMS has a positive relationship up to 0.5 wt% as the extra charge of the fluorine group extends the capacitance between the films; the addition of more than 0.5 wt% reduces the output due to aggregation of the filler in the matrix. A minimum power density of 4.68 μW cm^−2^ is obtained from the TENG and a maximum power density of 52 μW cm^−2^ from M-TENG (11 times greater than TENG). For practical applications, the fabricated M-TENG has been used for creating a generated voltage by simple human motion, such as tapping or walking, which is shown in Fig. 4m. In addition, for illuminating an 8-bit LCD, as illustrated in Fig. 4n the M-TENG has shown results in charging storage capacitors, as described in Fig. 4o, which indicates that it has potential as an excellent mechanical energy scavenging technology [83].

**Fig. 5:**
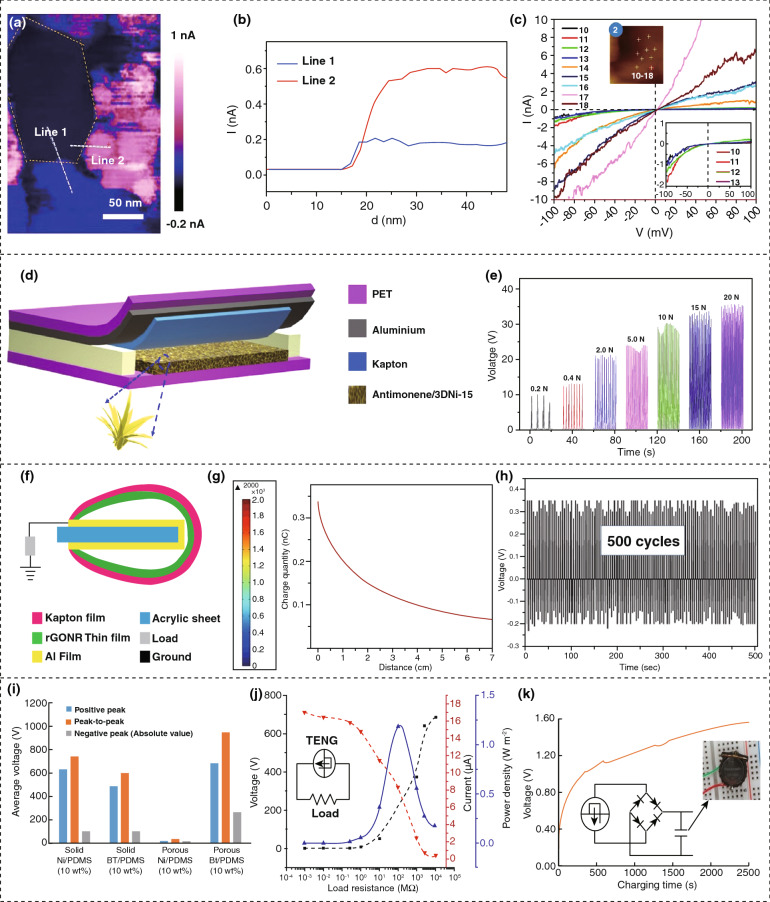
Schematics of structure and output performances of triboelectric nanogenerators. **a** Image of the adjacent MoS_2_ crystal divided into three grains from atomic force microscope and their current distribution in the three regions. **b** Cross-sectional current lines in the grains. **c** Spectrum of current–voltage characteristics of the grains 2. **d** Schematic of the triboelectric nanogenerator. **e** Rectified output voltage from the device. **f** Schematic of triboelectric nanogenerator with a single electrode based on reduced graphene oxide nanoribbons/ polyvinylidene fluoride. **g** Generated charges of Al foil, which decreases with the increasing gap between the film and foil. **h** Stable operation of triboelectric nanogenerator for 500 cycles. **i** Comparison of triboelectric generator voltage while fabricated by porous and solid nanocomposites. **j** Output voltage, current, and power. **k** Performance in powering a lithium coin cell. Figures reprinted with permission from: **a**, **b**, **c** Ref. [84], © 2018 Nature; **d**, **e** Ref. [85], © 2020, Elsevier; **g**, **h**, **i** Ref. [71], © 2016 Nature; **j**, **k**, **l** Ref. [72], © 2018 Nature

Rather than using dielectric materials, the gliding of a platinum-covered silicon AFM (atomic force microscope) tip as the external voltage source across multilayers of molybdenum disulfide (MoS_2_) leads to the creation of a TENG. In this case, triboelectricity is generated and MoS_2_ plays an important role in charge transfer [84]. Figure 5a shows three different triboelectric current responses of MoS_2_ grains where *I*_grain2_ > *I*_grain3_ > *I*_grain1_, and Fig. 5b illustrates the cross-sectional current of Fig. 5a. Figure 5c represents the voltage-current behavior of grain 2 and the magnified measurement of voltage-current spectra of grain 2 from points 10–13 are shown in the inset. The reason for the variation in values is due to the variation of surface charge in different grains of MoS_2_ since the bottom electrode tends to be oxidized. The external voltage supplied by the AFM tip helps the nanogenerator to increase the generated triboelectric current density, which can be used in many large-scale mechanical energy harvesting purposes by the integration of many such devices [84]. A core–shell-like structure consisting of metal Ni as core and metalene (antimonene nanodendrite) shell-based nanogenerator is fabricated that can produce triboelectricity and provide electrochemical energy storage [85]. This dual functionality offers an optimistic possibility for the polymers for energy harvesting and storage applications. Figure 5d shows a schematic of the fabricated nanogenerator, which is fabricated with metalene by electrochemical deposition technique (EDT). The fabricated device can act as a capacitor with a specific capacity of 1618.41 mAh. The generated rectified output voltage under the mechanical force in the range from 0.2 to 20 N is shown in Fig. 5e where a maximum voltage is found at 20 N [85].

Furthermore, thin-film reduced graphene oxide nanoribbons (rGONRs) incorporated within a polyvinylidene fluoride (PVDF) polymer have been applied in fabricating an arch-shaped TENG with a single electrode, as illustrated in Fig. 5f. The rGONRs exhibit charge negativity due to the larger diameter ratio, extreme edges, and electronegative oxygen-containing functional groups. This type of architecture that combines the surface roughness incorporated with the generating functional groups can improve the charge storage ability of the rGONRs/PVDF film [86]. The charge production on aluminum (Al) foil decreases with an increase in the gap between the rGONRs/PVDF film and aluminum (Al) foil, which is presented by Fig. 5g. This fabricated TENG has led to highly stable behavior after a stability test for 500 cycles, as illustrated in Fig. 5h, and this reliable behavior is important for applications in portable electronic devices [86]. A rapid fabrication technique with porous BaTiO_3_ or a polydimethylsiloxane (PDMS) nanocomposite-based TENG has been introduced, where the fabrication process is made more simple and effective [87]. A porous BaTiO_3_/PDMS-based nanogenerator was fabricated by microwave (MW) irradiation that can increase the output voltage by 83% and 130% for a positive and negative voltage, respectively. The resulting voltage from the TENG fabricated with a solid and porous nanocomposite is presented in Fig. 5i, which confirms the superiority of the porous nanocomposite. The voltage, current, and maximum power density at 100 MΩ load resistance are depicted in Fig. 5j. The fabricated device is capable of charging capacitors, rechargeable coin cells, and several hundred light-emitting diodes (LEDs). Figure 5k shows the capacitor charging efficiency and the TENG can charge 3 V lithium cells up to 1.6 V in 2500 s [87].

With additional investigation, such as utilizing a graphene/copper heterojunctions for electron transfer [88] and large-area graphene synthesis at the exterior side of the copper with the help of chemical vapor deposition (CVD) process [89], more stable and effective TENG fabrication processes are being developed. The functional nature of 2D nanomaterials such as graphene can avoid the oxidation of the Cu (copper) electrode at room temperature, which leads to higher device efficiency [88]. A chemical etching-free, residual polymer-free, and environmentally friendly method is used to grow graphene on copper by the CVD (chemical vapor deposition) process on the surface of transparent EVA (ethylene vinyl acetate) or PET (polyethylene terephthalate) [89]. The upcoming research on the synthesis and fabrication methods will develop this sector for the large-scale production of 2D nanomaterials, such as graphene, in an effective way. To concentrate on ambient energy sources and achieve a more efficient operation by the appropriate utilization of mechanical energy from both sides of the electrode, a robust thin film-based TENG array for scavenging bidirectional wind energy has been developed for self-powered devices [90]. The newly fabricated nanogenerator opens up a new way of thinking to utilize all available surrounding energies more effectively by developing a more suitable device architectural design. Achieving an overall improvement is a continual process for exploiting 2D nanomaterials for triboelectricity generation. The tunable properties and flexibilities of 2D nanomaterials are making TENG more efficient in mechanical energy conversion from small-scale vibrational motion to heavy sea waves [5]. There are some demerits in terms of material synthesis complexity, slow growth rates, and high costs. In addition, the storage and fabrication costs are a barrier in triboelectric nanogenerator research for the scientific community [85, 86]. The application areas of these fabricated devices are highly versatile and cut across many fields, including device structure, functional materials, fabrication process, device outputs, reliability. Applications of the reported work are summarized in Table 2.

**Table 2:** Comparative summary of reported work on triboelectric energy harvesting including device structure, functional materials, fabrication techniques, device outputs, reliability, and applications

Device structure	Functional materials	Fabrication methods	Device outputs/ Reliability	Applications	References
	TiO_2_ nanoparticles	Dispersion, chemical coating, electrospinning	*V*_oc_ = 115 V Efficiency = 92% Up to 3 cycle output remains 90%	Self-powered nanosystem, on-skin sensors, wearable devices	[80]
	GO dispersion	Curing, molding, demolding	*V*_oc_ = 123.1 V *J*_sc_ = 18.61 mA/m^2^ Power density = 4.97 Wm^−2^ Stable output up to 60 days	Bending monitoring, Pressure monitoring	[82]
	Fluorinated metal–organic framework	Dispersion, electro-spinning	*V*_oc_ = 550 V *I*_sc_ = 2.6 µA Power density = 121 µWcm^−2^	Energy source for LCDs and capacitors	[83]
	Antimonene, 3D Ni	EDT (electrochemical deposition technique)	*V*_oc_ = 54 V *I*_sc_ = 87 µA Power density = 6.85 µW cm^−2^ Much higher charging period	Integrated energy systems	[85]
	Reduced graphene oxide nanoribbons (rGONRs)	Drop casting technique	Output voltage = 0.35 V Shows stability up to 500 cycles	Energy harvesting	[86]
	BaTiO_3_, PDMS nanocomposite	Microwave irradiation	Maximum power density = 1.184 W m^−2^ Fabrication time is shorter	On-skin sensors, soft actuators	[87]
	Reduced graphene oxide (rGO)	Physical exfoliation, spin coating	*V*_output_ = 60 V, Maximum power density = 91.9 mW m^−2^ Stable up to 10,000 bending cycles	Power electronics	[88]
	Graphene, PDMS	CVD (chemical vapor deposition)	*V*_output_ = 22 V *J*_sc_ = 0.075 µA cm^−2^ Copper and R2R process can be reused, environmentally friendly	Graphene production industries	[89]
	Ti, Kapton film, Al	Laser cutting method	*V*_oc_ = 342 V *I*_sc_ = 66 µA Output power = 3.4 mW	Harvesting bidirectional energies, wireless sensors	[90]

### Piezoelectric Power Generation

In a similar way to triboelectric power generation, the *piezoelectric effect* is a method where mechanical energy can be converted to electric energy. 2D nanomaterials are now being extensively used for both direct current (DC) and alternating current (AC) piezoelectric power generation. 2D nanomaterials exhibit excellent piezoelectric properties in energy scavenging, while they do not exhibit such properties in their bulk state [91]. In general, non-centrosymmetricity is a required property for a material to be piezoelectric because the electrical polarization is associated with it during the coupling of mechanical and electrical behaviors [92]. The device structure with fabrication methods and functional materials, outputs, and applications of the reported piezoelectric power generating devices based on 2D nanomaterials is summarized in Table 3.

**Table 3:** Comparative summary of reported work on piezoelectric energy harvesting including device structure, functional materials, fabrication techniques, device outputs, and applications

Device structure	Fabrication	Functional materials	Outputs	Applications	References
	Thermal evaporation	ZnO nanosheets	*V*_output_ = 0.44 V *J*_sc_ = 6.5 µA cm^−2^	Direct power generation	[93]
	Beam evaporation	ZnO nanosheets	*V*_output_ = 0.9 V *J*_sc_ = 16.5 µA cm^−2^ Power density = 600 nW cm^−2^	Mechanical sensors, portable electronics	[94]
	CVD	MoS_2_	*V*_oc_ = 15 mV *I*_sc_ = 20 pA Power density = 2 mW m^−2^ Efficiency = 5.08% (mechanical–electrical)	Nanodevices, bioprobes, stretchable/tunable electronics	[95]
	Spray coating	Graphene films	Resistance recovery = 0–50% Gauge factor = 6.4 to 12.06	Monitoring body movement and muscle contracting	[97]
	3D printing	PTFE PVDF	Output voltage = 12.1–51.6 V, average power = 1.04 W	Bridging networks, powering portable electronic devices	[98]

**Fig. 6:**
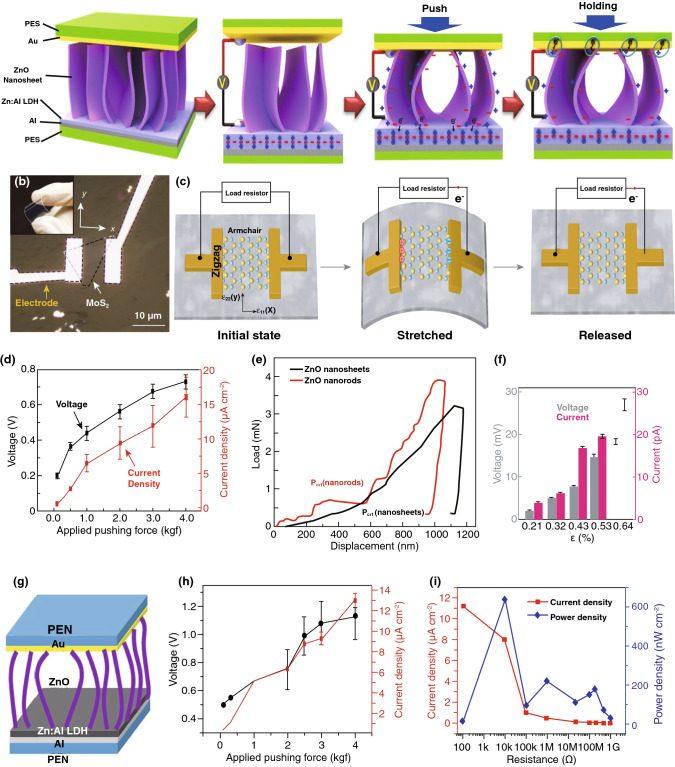
Device structure, working mechanism, and performance of piezoelectric energy generation. **a** A two-dimensional ZnO-based nanogenerator fabricated nano-clay-layered heterojunction and its working principle for energy scavenging. **b** A typical flexible nanogenerator based on single-layered MoS_2_ nanoflake, including device image in inset. **c** Operating mechanism of the piezoelectric. **d** Resulting voltage and current according to the external vertical. **e** Plot of load–displacement of nanorods and nanosheets derived from ZnO. **f** Relation between the piezoelectric response and applied external strain. **g** Three-dimensional structure of ZnO nanosheet-based piezoelectric nanogenerator. **h** Effect of applied force on voltage and current. **i** Piezoelectric current along with power densities of the device with an external circuit resistance. Figures reprinted with permission from: **a**, **d**, **e** Ref. [93], © 2013 Nature; **b**, **c**, **f** Ref. [94], © 2020 Elsevier; **g**, **h**, **i** Ref. [95], © 2014 Nature

A piezoelectric nanogenerator (PENG) has been fabricated using a 2D ZnO nanosheet, with its piezoelectric DC power generation process shown in Fig. 6a [93]. In this case, a gold (Au)-coated polyethersulphone (PES) has been used for the upper electrode and aluminum as the lower electrode. A 2D ZnO nanosheet/anionic nano-clay-layered heterojunction is synthesized amongst the two electrodes. When an external force acts on these nanosheets, it creates a positive potential on the outside of the expanded nanosheets, generating the negative electrical potential on the inside of the shrunk nanosheets, as shown in Fig. 6a. During the holding time, the positive potential of the nanosheets starts to decrease by attracting electrons, which are generated from the upper Au electrode connected to the compressed nanosheets until the potential becomes neutral when the force is released. As a result, the device generates only a DC pulse in every pushing and releasing period. The resulting current density and voltage depend on the level of the applied pushing force, which is represented in Fig. 6d where it can be found that a greater degree of force leads to a higher output. However, it is also necessary to consider the mechanical stability during operation of the fabricated PENG, and therefore, it is subjected to mechanical testing for comparison of the load–displacement curve between nanorods and nanosheets, as depicted in Fig. 6e [93]. Using a similar mechanism, a ZnO nanosheet-based PENG is represented in Fig. 6g [94]. The density of the nanosheets decreases the aggregation of freestanding of ZnO residues, which leads to an improved output performance. In this case, the voltage is increased up to 1.15 V, which is shown in Fig. 6h. It also shows an excellent power density of 600 nW cm^−2^ at 10 kΩ resistance, as illustrated in Fig. 6i. The fabricated device also provides a persistent output without any degradation in the output for up to 4000 cycles, indicating the durability of the device [94].

A single atomically thin-layered MoS_2_ has been investigated for piezoelectric harvesting and fabricated as a flexible PENG. Figure 6b displays a diagram of the fabricated device, and the working mechanism is represented by Fig. 6c [95]. When the PENG is stretched by applying a mechanical strain, both a positive and negative induction of the piezoelectric polarization charges occurs at the edges of the MoS_2_ adjacent to the electrodes, as shown in Fig. 6c. This results in a current flow through the external circuits. Similarly, during the relaxation period, electrons flow along with the load circuit in an alternating direction, as indicated by an arrow in Fig. 6c. The resulting voltage and current from the device are directly related to the maximum value of the strain that is applied, which is presented in Fig. 6f, and the fabricated PENG can easily sense the level of strain applied to it [95].

A new organic–inorganic ferroelectric perovskite material, known as Me_3_NCH_2_ClMnCl_3_ (TMCM-MnCl_3_), has been discovered with elevated piezoelectric properties that can be further modified by replacing with a new element or new molecular structure according to the application, which is attractive for nanosystems and self-powered devices [96]. This opens the door to solve the issues of rigidity and toxicity of piezoelectric materials to make them soft, lightweight, and biofriendly for applications in health monitoring sensors. For practical applications, piezoelectric nanogenerators are of interest when fabricated with nanomaterials that make them highly sensitive and lightweight. A stretchable graphene thin film-based yarn sensor having adjustable piezoelectric properties has been fabricated by combining a graphene oxide (GO) layer covering the polyester (PE) wound spandex yarns, which can provide any electrical signal from the small movement of human motion [97]. Here, the electron transport behavior of the graphene film is changed by varying the stretch level, which leads to a better sensitivity of the sensor. Hybrid power generating systems, such as a PENG coupled with a TENG, has been introduced recently for achieving better efficiency and flexibility of multi-level scavenging capability [98]. The fabricated hybrid nanogenerator can generate piezoelectrical power from the self-oscillating response of the impact-induced friction and triboelectric power is due to the effect of impact-induced friction directly. Effective piezoelectric generation depends on the behavior of the materials, where non-symmetricity is one of the most important. In this case, 2D nanomaterials such as single-layered, atomically thinned materials are capable of exhibiting non-symmetric properties that are not possible in the bulk state. Other properties such as flexibility, mechanical stability, conductivity can also be further improved by different methods. In terms of demerits, the piezoelectric materials can be toxic and fragile, which can be a challenge for environmental and health monitoring applications [96]. In addition, a vulnerable structure is not ideal for mechanical operations during piezoelectric generation.

### Thermoelectric Power Generation

**Fig. 7:**
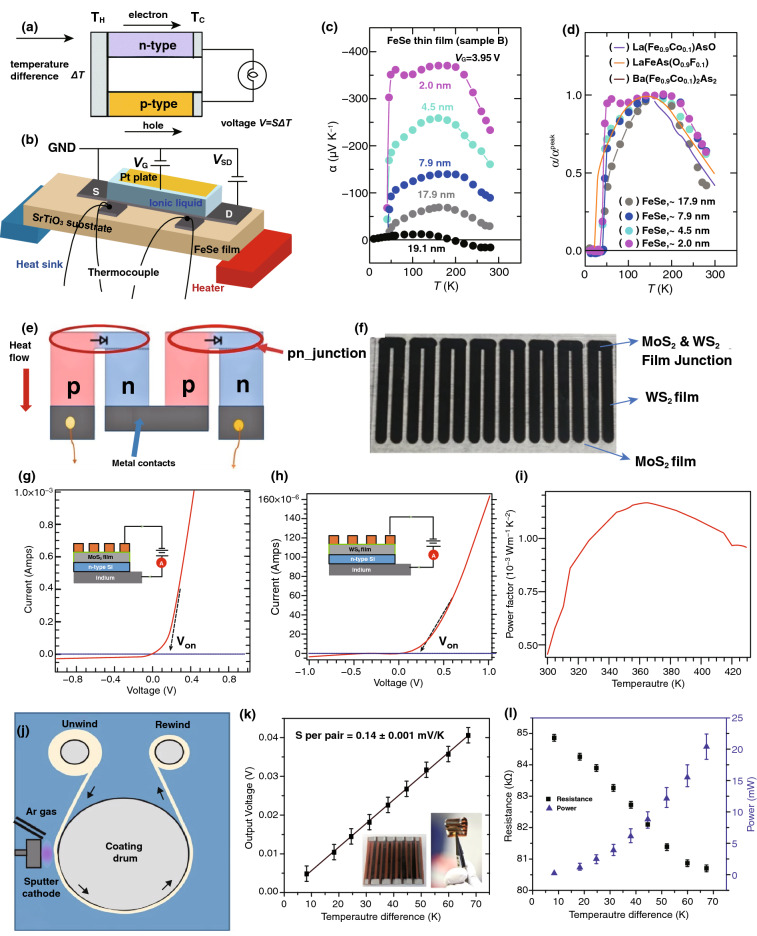
Thermoelectric device structure, the working principle, and output performances of thermoelectric devices. **a** Mechanism of thermoelectric power generation due to Seebeck effect. **b** Device architecture for thermoelectric measurement. **c** Curve of Seebeck coefficient versus temperature. **d** Peak value of Seebeck coefficient according to temperature. **e** Thermoelectric nanogenerator based on p–n junction. **f** WS_2_ planar film as n-leg, while depositing in the fabrication process. **g** Current density–voltage characteristics of MoS_2_. **h** Current density–voltage characteristics of WS_2_. **i** Power factor of the MoS_2_ device. **j** Roll-to-roll arrangement for deposition on substrate. **k** Output voltage vs. temperature plots. **l** Plot of resistance and output power with changing temperature. Figures reprinted with permission from: **a** Ref. [101], © 2017, Royal Society of Chemistry; **b**, **c**, **d** Ref. [102], © 2019 Nature; **e**, **f**, **g**, **h**, **i** Ref. [103], © 2020 Nature; **j**, **k**, **l** Ref. [87], © 2019 Nature

A convenient way of utilizing ambient and low-grade waste heat from industrial processes is to generate electrical energy by employing thermoelectric (TE) devices through a process termed *thermoelectric* power generation. Due to their excellent thermal conductivity, 2D nanomaterials are widely used for the fabrication of TE devices. However, the thermoelectric device performance is closely dependent in accordance with the thermoelectric figure of merit *ZT* where *ZT* = *S*^*2*^*σ*/*k*_total_; *S* denotes the Seebeck coefficient, *σ* symbolizes the electric conductivity, and *k*_total_ refers to the total thermal conductivity [99]. In thermal energy scavenging, there can be four types of TE devices, which include the uncoupled thermal, thermoacoustic coupled, thermoelectric coupled, and the thermal and optical coupled devices; these are based on different materials, techniques for their synthesis, and application requirements [100]. A schematic of the mechanism of a thermoelectric generator is shown in Fig. 7a, where p-type and n-type semiconducting materials create a junction between them. For thermoelectric power generation in thermocouples, charge carriers are transported through the junction due to the temperature difference, ΔT (the Seebeck effect), which is the basic operational mode of thermoelectric nanogenerators [101]. High electrical conductivity and Seebeck coefficient can extensively increase the *ZT*, which is a crucial issue for obtaining higher outputs during the thermoelectric process.

2D nanomaterials are considered to be attractive compared to their bulk equivalent due to their density of states (DOS), which has an extensive correlation with the Seebeck coefficient, and can increase the device efficiency and *ZT* significantly [102]. A fabricated device is illustrated in Fig. 7b. The output thermoelectric coefficients depend on temperature as shown in Fig. 7c where it is found that the peak value of α is found at 200 K. Figure 7d shows the effects of thickness on the thermoelectric coefficient, where it can be seen that the thermoelectric coefficient increases due to the film being made thinner and is larger than the material in a bulk state. Thin films give higher normalized α^peak^ (peak value of the electrical conductivity) values [102].

Figure 7e is a schematic of a p–n junction-based thermoelectric generator (TEG), which is fabricated with RF (radio frequency) magnetron sputtering of MoS_2_ along with the WS_2_ thin films on the surface of a glass substrate, wherein the p-type or else n-type planar-patterned p–n junction is placed to the hot side of the device and the electrodes are at the cold side [103]. A clearer view of the MoS_2_ and WS_2_ films and the p–n junction of the fabricated TEG device are illustrated in Fig. 7f, which shows that the electrodes are contacted with planar legs. The current–voltage (*I–V*) characteristic curve behavior of the MoS_2_- and WS_2_-based TEG at room temperature, with an inset showing a schematic arrangement for measurement, are presented in Fig. 7g, h, respectively, displaying the start of increasing current at the turn-on voltage (*V*_on_) of 0.2 V. In addition, the power factor of the MoS_2_-based TEG is shown in Fig. 7i from where the highest calculated power factor is 1.15 × 10^–3^ W m^−1^ K^−2^ and is achieved at 370 K [103].

For fabricating TEG devices, various techniques are being developed according to the materials being used and the desired sector of applications. A physical vapor deposition (PVD) process with a roll-to-roll (R2R) process for high-throughput fabrication of Bi_2_Te_3_-/GeTe-based flexible TEGs has been investigated for thermoelectric power generation [104]. The R2R setup of the manufacturing method is represented in Fig. 7j, where the flexible substrate passes from an unwound spool to a rewound spool through the deposition area of the coating drum. The voltage output of each thermoelectric couples of the Bi_2_Te_3_/GeTe, along with the Seebeck coefficient per each couple, leads to a value of 140 ± 1 μV K^−1^, as illustrated in Fig. 7k [104]. The inset shows a schematic of the device. Figure 7l provides measurements of the resistance and power with the changing temperature of the generator, which indicates that the output power improves by the elevation of temperature difference.

A sandwich structure of a graphene van der Waals heterostructure with multilayer transition metal dichalcogenides fabricated between graphene electrodes could scavenge waste heat at an efficiency of 7–8% at 400 K [105]. The extremely low cross-plane thermal conductance of 2D nanomaterials and thermionic emissions beyond the Schottky barrier (SB) between graphene and other 2D nanomaterials have led to the device showing greater performance than traditional TEG devices [105]. A self-powered device was fabricated with graphene-eco flex nanocomposite, operating based on the photo-thermoelectric (PTE) effect that combines photo-thermal (light to heat) and the thermoelectric (heat to electrical) effect simultaneously, which provides a power density of 36.1 mW cm^−2^ and excellent stability [106]. It is a highly convenient way to use graphene as a conducting filler in graphene–polymer-based strain sensors because of their excellent electron-transport property, and the TEG device facilitates strain measurements without any external power supply while also not harming environment. To increase the figure of merit (*ZT*) of the thermoelectric, materials undergo modification such as chemical modification, reducing thickness, defect engineering, and strain engineering for effective power generation through thermoelectric devices [101]. The reported work on thermoelectric power generation with device structure, functional materials, fabrication process, outputs, and applications of the thermoelectric devices is summarized in Table 4.

**Table 4:** Comparative summary of reported work on thermoelectric energy scavenging including device structure, functional materials, fabrication techniques, and device outputs

Device structure	Functional materials	Fabrication methods	Device outputs/ reliability	Applications	References
	FeSe film	EDL (electron double layer) laser cutting	Power Factor = 260 μW cm^−1^ K^−2^ Superconductive in ultra-thin stage	To develop new research on 2D nanomaterials for multi-functionality	[102]
	MoS_2_ film WS_2_ film	Shadow masking	Thermoelectric figure of merit = 1.98 Seebeck coefficient = 466.34 µV K^−1^	Moderate waste heat harvesting	[103]
	Graphene TMDs	-	Waste heat harvesting efficiencies 7–8% at 400 K, and up to 20% at 500 K	Harvesting waste heat	[105]
	Graphene polymer	Vacuum filtration	Power density = 36.1 mW cm^−2^, strain sensitivity = − 0.056 In(nA)/%, Sensing resolution = 0.5%, response and recovery time = 0.6 s	Implantable health monitoring devices, smart wearable devices	[106]

### Pyroelectric Power Generation

Pyroelectric power generation is another way of utilizing waste heat energy by exploiting the pyroelectric effect. The main difference between the thermoelectric and pyroelectric process is that temperature gradients are used for the thermoelectric process; a fluctuation of temperature is required for pyroelectric materials [107]. When a pyroelectric is heated, the polarization of the material decreases as dipoles lose their orientation, thereby creating free charges on the electrode interface. When the pyroelectric device is under an open-circuit condition, the charges remain on the interfaces to create an electric field. On applying a short circuit or electrical load, the charges are able to flow in an external circuit to create a current. Similarly, during cooling, the polarization of the material increases, and charges flow in the opposite direction [108]. The main advantage is that the thermoelectric materials can operate with large thermodynamic efficiency without any requirement of a large heat sink as used in the thermoelectric process to achieve a high-temperature gradient [109]. The performance of pyroelectric nanogenerator, figure-of-merit (*F*_E_) is symbolized as, *F*_E_ = *p*^*2*^Δ*T*/*C*_E_*Ɛ*_33_; here, *p* denotes the pyroelectric coefficient of materials, Δ*T* indicates the increment of temperature, *C*_E_ refers to the heat capacity specified by volume, and *Ɛ*_33_ denotes the permittivity [110].

2D nanomaterials are materials of interest because they can exhibit a change in polarization under external temperature changes or mechanical forces. Considering that all the pyroelectric materials are also piezoelectric, they have the potential to be used for thermal and mechanical scavenging [108]. The atomically thin 2D nanomaterials, such as thin films, can extend the pyroelectric output because of their extremely low thickness by increasing the rate of temperature change and temperature difference. A hybrid nanogenerator is fabricated that performs both piezoelectric and pyroelectric power generation with solar power conversion, which increases the output significantly [111]. Thus, the materials can be used to fabricate multi-functional devices for use for a range of possible sources of non-conventional energies.

**Fig. 8:**
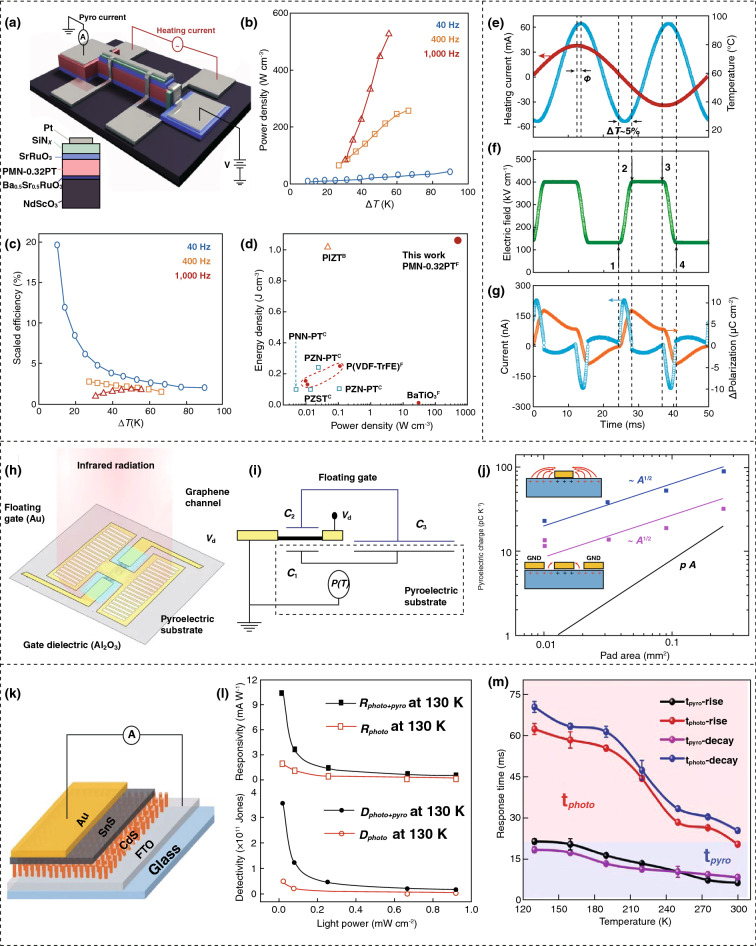
Device fabrication and output performances of pyroelectric nanogenerators. **a** Illustration of device structure. **b** Power density. **c** Scaled efficiency of the device. **d** Comparison of this work with other reports. **e** Application of Ericsson cycle with varying temperature (blue line) and electric field (red line). **f** Implementing a periodic current across the relaxor. **g** Output current and resulting polarization in response to varying temperatures. **h** Schematic of device with a graphene channel and floating gate. **i** Device circuit diagram. **j** Accumulated pyroelectric charge according to pad area. **k** Schematic structural diagram. **l** Device responsivity with detectivity. **m** Responding period of the pyro and photoelectric effect. Figures reprinted with permission from: **a**, **b**, **c**, **d**, **e**, **f**, **g** Ref. [112], © 2018 Nature; **h**, **i**, **j** Ref. [113], © 2017 Nature; **k**, **l**, **m** Ref. [114], © 2020 Wiley. (Color figure online)

A pyroelectric energy scavenging device is shown in Fig. 8a, which is fabricated with a relaxor ferroelectric thin film and operated using a pyroelectric Ericsson cycle with a high energy density of 1.06 J cm^−3^, a power density of 526 W cm^−3^, and high efficiency of 19% of Carnot [112]. The output power density and scaled efficiency are presented in Fig. 8b, c, with the function of frequency that shows the highest power density of 526 W cm^−3^ at a temperature of 56 K, along with the frequency of 1000 Hz, and the largest scaled efficiency of 19% at a temperature 10 K, along with the frequency of 40 Hz. Figure 8d is a comparison between the energy and the power density of this proposed generator with prior work, which indicates that an improved pyroelectric coefficient is obtained by providing better power and energy density. Figure 8e presents the effect of applying an external sinusoidal current to the device that heats the heterojunction via Joule heating where the red and blue lines denote the heating and temperature of the heterostructure. Figure 8f depicts the applied periodic electric field *E*(*t*) that triggers the isothermal polarization and depolarization when the temperature gets to its extrema. The change of polarization Δ*P*(*t*) (orange line) is obtained by integrating the total background pyroelectric and dielectric (blue spikes) current with varying temperatures and electric field, as shown in Fig. 8g [112].

A graphene bolometer has been fabricated using a LiNbO_3_ pyroelectric substrate with a graphene channel and floating gate, as illustrated in Fig. 8h, for the detection of thermal infrared (IR) radiation level at room temperature [113]. Figure 8i represents the circuit diagram of the fabricated device which has two terminals and changes the resistance according to the temperature. The total current flowing through the probe during a complete temperature-ramp cycle is dependent on the pad area (PA), as shown in Fig. 8j, which indicates that small pixels of dense arrays can provide elevated device efficiency. An increase in the cryogenic light detection efficiency is achieved by integrating the photoelectric effect with the pyroelectric effect based on a unique SnS/CdS heterojunction photodetector. The device structure is illustrated in Fig. 8k [114]. For evaluating the fabricated device, a responsivity of 10.4 mA W^−1^ along with the detectivity of 3.56 × 10^11^ Jones at 130 K is achieved, as shown in Fig. 8l, which indicates elevated device performance, especially at low temperatures. The response time of the photoelectric and pyroelectric effect is illustrated in Fig. 8m, which indicates that the device also has a short response time with a high output current.

A sunlight-triggered pyroelectric nanogenerator (S-PENG) has been fabricated when a reduced graphene oxide (rGO) and polyethyleneimine (PEI) layer are deposited on a polarized polyvinylidene fluoride (PVDF) film as formed in a sandwiched architecture with Ag electrodes. The maximum output power from the device is 21.3 mW m^−2^ that can power a fitness monitoring electronic device [115]. There are some limitations, such as low efficiency because of synchronized electron extraction at a low frequency of operation, especially when compared to other effective harvesting energies such as higher frequency mechanical harvesters and photovoltaic cells, and they are bulky in size [111, 115]. However, pyroelectric devices can be used as a solid-state cooler as the applied field can cause the dipoles to regain their initial states. In addition, the pyroelectric nanogenerator is capable of hybridizing with other power generation techniques, such as piezoelectric or solar power to utilize mechanical and solar power along with the waste heat harvesting from ambient surroundings with a single device [111]. The device structure with fabrication methods and functional materials with the output and applications of the reported fabricated devices is summarized in Table 5.

**Table 5:** Comparative summary of reported work on pyroelectric energy harvesting including device structure, functional materials, fabrication techniques, and device outputs

Device structure	Functional materials	Fabrication methods	Device outputs	Applications	References
	Ba_0.5_Sr_0.5_RuO_3_, NdScO_3_	Pulsed-laser deposition, plasma-enhanced CVD	Maximum energy density = 1.06 J cm^−3^, Power density = 526 W cm^−3^ per cycle, Scaled efficiency = 19%	Pyroelectric energy harvesting from low-grade heat	[112]
	LiNbO_3_ Graphene	CVD	Temperature coefficient of resistance, TCR = 900 K^−1^, temperature variation as lower to 15 µK	Highly sensitive spectroscopy in MIR and far-IR	[113]
	SnS, CdS	Thermal evaporation	Power density = 0.08 mW cm^−2^, Responsitivity = 10.4 mA W^−1^, Detectivity = 3.56 × 10^11^, Response time = 30 ms	High-performance cryogenic photodetectors	[114]
	rGO-PEI, polarized PVDF	Vacuum drying	Power density = 21.3 m Wm^−2^, can charge a wearable health kit within 1 h	Sunlight-triggered pyroelectric power generation for practical life electronic devices	[115]

### Osmotic Power Generation

Other forms of energy such as chemical energy can be harvested through techniques such as osmotic power generation, fuel cells, and zinc-/lithium-ion batteries to obtain electrical energy using 2D nanomaterials [116, 117]. Among these methodologies, in this review osmotic power generation will now be summarized. *Osmotic* power generation, also known as blue energy, refers to a promising technique where a different osmotic pressure between saltwater and freshwater is used to generate electrical power by a reverse electrodialysis (RED) process [118]. When an electrolyte is pushed through narrow pores with the applied osmotic pressure generated due to a pressure gradient [119] or salt concentration gradient [120], an electrolytic potential is produced. 2D nanomaterials are more favorable for such applications as they are atomically thin and have good electrochemical properties such as higher ion transportation rate and better cation selectivity for osmotic power generation [121]. Due to the chemical reaction generating power, the surface area and surface activity of 2D nanomaterials play an important role in osmotic power generation.

**Fig. 9:**
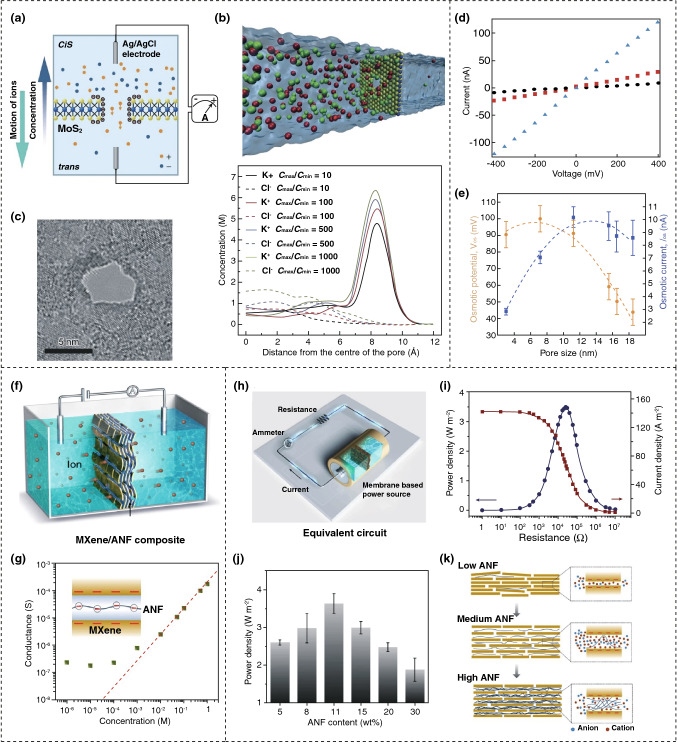
Working mechanism of osmotic power generation. **a** Arrangement of module by membrane separating the solutions with different density. **b** A simulated distribution of ions in solution. **c** MoS_2_ nanopore membrane, drilled with transmission electron microscope with 5 nm diameter. **d** Output current, and voltage according to the pore size. **e** Osmotic voltage and current according to pore size. **f** Schematic of the device arrangement. **g** Calculation of conduction with the varying concentrations. **h** Setup for transporting generated power to external load circuit. **i** Generated power density by the membrane. **j** Effect of aramid nano-fiber concentration on output power. **k** Illustration of aramid nano-fiber fraction on overall energy conversion. Figures reprinted with permission from: **a**, **b**, **c**, **d**, **e** Ref. [122], © 2016 Nature; **f**, **g**, **h**, **i**, **j**, **k** Ref. [13], © 2019 Nature

An osmotic power generator is introduced, where two reservoirs with a different concentration of potassium chloride (KCl) are separated with a MoS_2_ membrane with nanopores, as illustrated in Fig. 9a [122]. The upper part of Fig. 9b is a simulation of the molecular-dynamics describing the movement of ions within the nanopore in the analyte. The lower graph represents the concentration of ions with its distance from the core of the nanopore. The diameter of this nanopore is varied 2-25 nm in the investigation. Figure 9c is the TEM image of the membrane, a pore in the middle indicating a pore size of ~ 5 nm. Figure 9d presents the voltage and current of the membrane at different diameters at a fixed concentration of 1 M KCl. It is observed that saturated conductance is found at lower concentrations, but with increasing concentration, the conductance is increased. The osmotic power generation according to the pore size is illustrated in Fig. 9e, where it can be seen that the membranes with smaller pores are more efficient due to greater ion selectivity.

An MXene-based nanofiber composite membrane for osmotic energy scavenging has been demonstrated, which can increase the output by utilizing the space charge of the nanofiber for improved ion diffusion and better energy conversion [13]. Using both river water and seawater, the generator can provide a power density of 4.1 Wm^−2^. Figure 9f is a schematic drawing of the device configuration for determining the transmembrane ion transfer. The relationship between the conductance and the concentration of potassium chloride (KCl) in the analyte is illustrated in Fig. 9g, where decreasing the concentration is liable for reduced conductance. The simulated ion transfer is shown in the inset. For utilizing the converted energy in practical applications, the complete circuit diagram with the load and generator is presented in Fig. 9h, and the effect of resistance to the load circuit on power and current density is depicted in Fig. 9i indicating a lower current density with higher resistance since the resistance is restricting current flow. At a low load resistance, the current is high, but the voltage is low; therefore, there is an optimum where both voltage (*V*) and current (*I*) are sufficiently large to create a maximum power (*P*), since *P* = *VI*. With an increasing weight of ANF (aramid nanofiber), the power density becomes a maximum at 11% (wt%) and then decreases again, and the relation is shown in Fig. 9j. This is due to the interaction of ANF that extends the space between the flakes, which leads to an increase in ion flux through the nanochannel. Figure 9k shows the effect of the weight ratio of ANF on overall energy scavenging efficiency, where the efficiency is decreased with a high amount of ANF due to the blocking capability of ANF in the 2D channel. The major challenges in osmotic power generation are the selectiveness of ions and to maintain appropriate concentration gradients [122]. This method can be highly productive because of the abundant availability of seawater and more comprehensive research is required to establish a highly efficient osmotic power generation plant, and 2D materials can play a significant role in this area.

### 2D Nanomaterial-Based Self-Charging Supercapacitors

Conventional energy systems are lacking in the case of continuous supply networks or recharging batteries and capacitors frequently. This can cause problems, such as a discontinuous power supply, because of their dependency on charging batteries and capacitors with external sources, which can be difficult in bad weather conditions or during mass natural disasters. These problems can be overcome by using a nanogenerator-based energy harvester with inbuilt energy storage systems. The self-charging supercapacitor power cell (SCSPC) can harvest a range of possible types of sustainable energy resources to recharge themselves without any external power supply. 2D nanomaterial-based self-charging supercapacitors based on different architectures of separators and electrolytes include electrospun nanofibrous piezoelectric separator including an ion gel electrolyte [123], two graphene sheets creating a sandwiched structure containing porous PVDF-based ionic liquids inside [124], and symmetric electrodes with a piezo-electrolyte [125]. The self-charging mechanism lies in the piezo-electrochemical conversion process, which can be understood with piezo-electrochemical spectroscopic measurements [127]. The introduction of the self-charging mechanism has led to the fabrication of a self-chargeable flexible solid-state supercapacitor (FSSSC) [128], a self-charging sodium-ion battery [129], and a highly stretchable self-chargeable supercapacitor [130], which facilitates opportunities to develop wearable and flexible electronic devices that can be charged without any external power sources. Recent developments in self-charging supercapacitor-based power cells have proven to be more effective in finding a higher charging voltage, such as a porous PVDF device with a specific device capacitance of 31.63 mF cm^−2^ [124]. A MoSe_2_ solid-state supercapacitor based on the electrospun nanofibrous piezoelectric separator and ionogel electrolyte provides a self-charging voltage up to 708 mV, with a power density of 268.91 µW cm^−2^ [123]. Recently, a novel flexible solid-state self-charging supercapacitor has been fabricated with Co-doped Fe_2_O_3_ grown on the activated carbon cloth (Co-Fe_2_O_3_@ACC) and BaTiO_3_ piezoelectric particles mixed with a solid electrolyte system poly(vinyl alcohol) (PVA) complexed with potassium chloride (KCl) (PVA-KCl-BaTiO_3_) as symmetric electrodes and electrolyte, respectively [125]. The fabricated device has excellent flexibility and charging capability with significant electrochemical properties that can synchronously harvest and store energy. It can charge up to 120 mV by simple bending in 7 min at a low frequency of 1 Hz, which can be used in portable electronics applications.

**Fig. 10:**
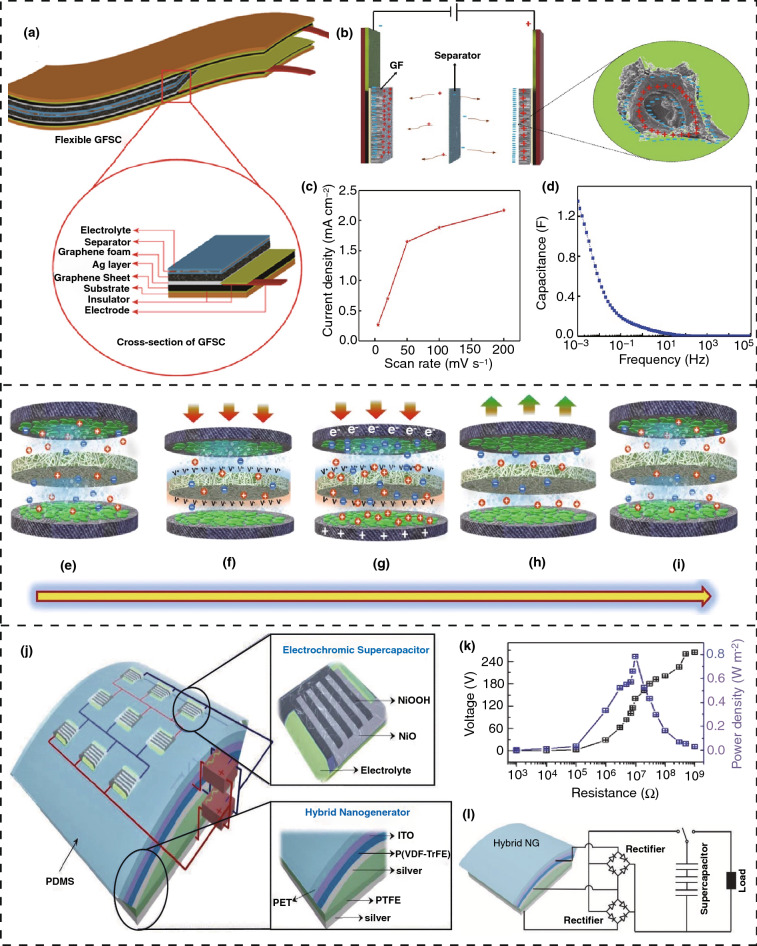
Self-charging mechanism and supercapacitor performances. **a** Cross section of graphene foam supercapacitor. **b** Electrochemical reactions of the electrolyte ions on the surface of the electrodes. **c** Current density plot with the scan rate. **d** Capacitance of the fabricated supercapacitor. **e** Initial state of the siloxene supercapacitor. **f** Migrating electrolyte ions toward the electrodes due to the piezoelectric effect. **g** The new equilibrium state after the piezoelectrochemical reaction. **h** Effect of removing the compression; the disappearance of piezoelectric field. **i** Returning to the initial state after a complete self-charging cycle. **j** Schematic of the compact power device with the electrochromic supercapacitors and hybrid nanogenerator. **k** External load effect on the output voltage and power density. **l** The circuit diagram of rectification of the generated power from the device. Figures reprinted with permission from: **a**, **b**, **c**, **d** Ref. [126], © 2021 Elsevier; **e**, **f**, **g**, **h**, **i** Ref. [127], © 2020 Nature; **j**, **k**, **l** Ref. [131], © 2016 Wiley

A flexible graphene–Ag–graphene foam electrodes based self-charging supercapacitor have been fabricated as an energy harvesting and storage technology [126]. The graphene foam-based supercapacitor (GFSC) shows excellent electrochemical and super capacitance properties with a current density of 0.67 mA cm^−2^ and capacitance of 38 mF cm^−2^. A schematic of the device is shown in Fig. 10a, where the graphene sheet, Ag layer, graphene foam, electrolyte, and separator are stacked on the substrate sequentially. The H^+^ and PO_4_^3−^ ions from H_3_ PO_4_ are absorbed on the cathode and anode electrodes, respectively, during the charging steps, as shown in Fig. 10b. The adsorption of ions by the electrodes due to the electrostatic and Faradaic reaction results in the formation of an electrical double layer along the high surface area of graphene foam. The rate of adsorption increases because of the lower hydrophobicity of the high surface area of the graphene foam. Similarly, during the discharging stage, the ions move from the electrodes to the electrolyte due to the absence of an electric field. The current density of the supercapacitor increased with an increasing scan rate, as presented in Fig. 10c, which is important to analyze the predominant diffusion mechanism governing the reaction of ions from electrolytes to GFSC electrodes surface. The specific capacitance of the supercapacitor is shown in Fig. 10d, which is of interest in practical applications. The fabricated device has a specific capacitance of 1.3 F at 1 MHz. The stability of the device is also high with 68% retention after 25,000 charging/discharging cycles. In addition, the device was integrated with a photovoltaic cell to obtain a self-charging power pack and was used for continuous power supply to a wearable pH sensor [126].

Generally, the most widely studied self-charging supercapacitors are based on piezoelectric conversion [123–125] where the piezo-electrochemical effect is used for the energy conversion. To investigate this mechanism, a piezoelectric-based self-charging supercapacitor power cell (SCSPC) has been fabricated where siloxene sheets work as the electrodes and siloxene-based piezo fiber works as the separator that is immobilized with an ionogel electrolyte [127]. The measurements are completed with the help of piezo-electrochemical spectroscopy (PESC) to directly probe the piezo-electrochemical effect of the siloxene SCSPC. The physical structure of the fabricated device is as in Fig. 10e in which the electrodes are made of siloxene-coated carbon cloth and ionogel-coated electrospun siloxene-PVDF piezo fiber as the separator. The working mechanism of the SCSPC can be explained through the piezo-electrochemical process at the interface of the siloxene electrodes and siloxene-PVDF piezo fiber, as shown from Fig. 10e–i. At the initial state, the electrolyte ions are homogeneously distributed into the whole space of the cell, as shown in Fig. 10e. When an external compressive force is applied, a positive and negative piezoelectric potential is generated by the electrolyte ions that will be accessed by the siloxene electrodes as displayed in Fig. 10f. This migration of charges at the electrodes increases the charge density near both electrodes and creates an electrical potential difference that leads the SCSPC to undergo a self-charging cycle, as demonstrated by Fig. 10g. During the self-charging process, the potential difference between the two electrodes will continue to increase (a certain level of charging voltage) as the migration of opposite charges will increase the charge density until the SCSPC eventually achieves a new chemical equilibrium. At the new equilibrium state, the allocation of electrolyte ions will equalize the generated piezoelectric field, leading to termination of the self-charging process. When the compressive force is removed, as indicated by Fig. 10h, the piezoelectric effect will disappear which leads to redistribution of the electrolyte ions as the device moves into an equilibrium state (Fig. 10i). At this stage, the device is in its initial state to start a new self-charging cycle. A maximum self-charging capacity of up to 207 mV is found for the device by applying various levels of compressive forces.

The integration of a hybrid nanogenerator (TENG and PENG) with a solid-state electrochromic supercapacitor has fabricated a device with an excellent efficiency to charge capacitors [131]. A schematic of the device is shown in Fig. 10j. The compact device is integrated with two components, where the first component is a solid-state electrochromic micro-supercapacitor (µ-SCs) array based on silver nanowires/nickel oxide (AgNWs/NiO) active electrode materials at the upper part of the fabricated device. The second component is a hybrid nanogenerator containing a TENG based on Ag/PTFE/Ag (silver/polytetrafluoroethylene/silver) and a PENG based on IPO/P(VDF-TrFE)/silver (indium tin oxide/ poly(vinylidene fluoride-co-trifluoroethylene)/silver), as shown in the figure to generate triboelectric and piezoelectric power simultaneously. The generated output voltage and power are closely related to the external resistance as their relationship is shown in Fig. 10k. The output voltage decreases after a certain value of resistance that is close to a value of 10^7^ Ω, due to impedance matching, and the output power density reaches a maximum of 0.8 W m^−2^ at the resistance of 10^7^ Ω. To charge the electrochromic µ-SCs, a rectifier bridge network is used, as shown in Fig. 10l. The fabricated device can also store charge having the maximum capacitance of 3.47 mF cm^−2^ with the electrochromic µ-SC exploiting Ag nanowires/NiO as electrode materials. In addition, it has an excellent stability of 80.7% after 10,000 cyclic self-charging operations. This compact device can be further improved for applications in interactive display devices, touchscreen monitors, and electronic tagging systems.

## Applications of 2D Nanomaterial-Based Energy Scavenging Devices

The applications of 2D nanomaterial-based energy scavenging devices include versatile sectors such as nanosystems, wireless/self-powered sensors, and embedded self-powered devices that can be used in environmental monitoring [132], health technologies [133], industries [134], and human interaction with machines [135]. The discovery of 2D nanomaterials is enabling scientists to design miniaturized devices that are highly sensitive to small human or surrounding environmental signals. The ability to achieve a degree of autonomy and self-powered functionality provides greater opportunities for maintenance-free wireless operation, providing a greater degree of convenience as compared to conventional powered devices. The hybridization of different energy scavenging systems with self-charging batteries or supercapacitors can reduce a dependency on conventional batteries and power sources drastically, especially for electronic devices running on low power.

**Fig. 11:**
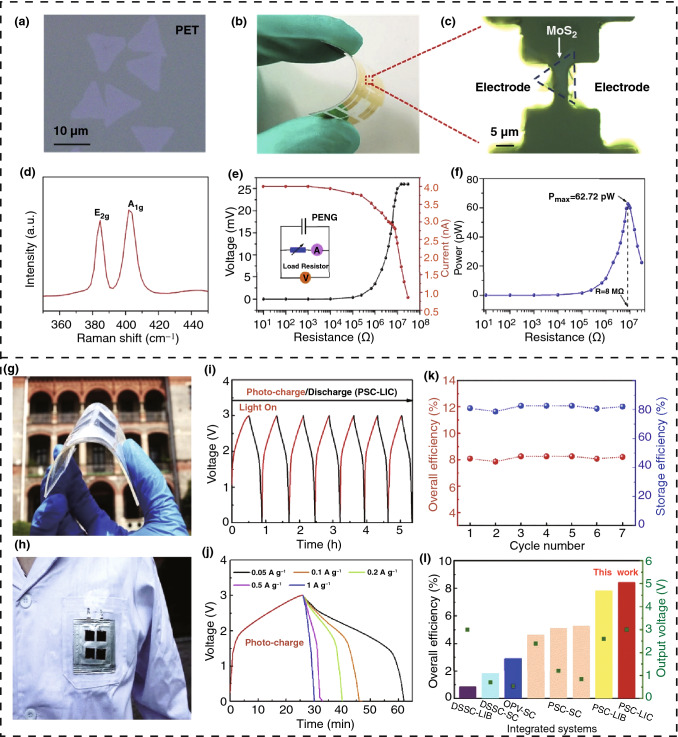
Nanogenerator-based self-powered sensors based on 2D nanomaterials. **a** Image of single-layer MoS_2_ flake via optical microscope. **b** Image of flexible piezoelectric nanogenerator. **c** Image of two electrode-based piezoelectric MoS_2_ device. **d** Raman spectrum of the flake. **e** Resulting voltage and current with measurement circuit in inset. **f** Measured power according to external resistance. **g** Image of flexible perovskite solar cell-oriented photo rechargeable lithium-ion device. **h** Image of device adhered to cloths for real-world application. **i** Voltage–time characteristics of the device. **j** Voltage–time characteristics. **k** Efficiencies of the device. **l** Comparison of device performance with other integrated systems. Figures reprinted with permission from: **a**, **b**, **c**, **d**, **e**, **f** Ref. [131], © 2019 Elsevier; **g**, **h**, **i**, **j**, **k** Ref. [135], © 2019 Elsevier

A flexible ammonia sensor with high efficiency at room temperature was fabricated with Au-MoS_2_ to exploit the piezoelectric properties of a single-layer MoS_2_ nanogenerator (PENG), whose image is displayed in Fig. 11b [132]. The fabricated sensor generates electrical power by responding to human motion, through piezoelectric power generation. Figure 11a shows a visual image of monolayer MoS_2_ film on the substrate. Figure 11c shows the internal structure of the fabricated device along with its double electrodes. A Raman spectrum of this fabricated device is presented in Fig. 11d, with two peaks with a difference of 20 cm^−1^ that confirms that the MoS_2_ flake has a mono-layered structure. The resulting voltage and current are represented in Fig. 11e, with load resistance from 10 Ω to 30 MΩ along with a fixed strain of 0.36% at an active 0.5 Hz frequency. The generated power of the device according to an applied load resistance is plotted in Fig. 11f, where the maximum value of power is 62.72 pW, when an external load of 8 MΩ is applied [132]. With further improvement, this sensor can be modified into a self-powered flexible gas sensing device in industrial sectors as well as scavenging human motion, and it can supply electrical power to several numbers of wearable electronic devices.

The utilization of solar energy scavenging techniques using a PSC (perovskite solar cell)-oriented photo-rechargeable lithium-ion capacitor for developing a self-powered strain sensor was fabricated with the capability of supplying up to 3 V with an efficiency of 8.41% [136]. Figure 11g is an image of the photo-rechargeable hybrid capacitor with an applied bending force. Figure 11h shows the practical use in sensing human motion attached to clothing. Figure 11i shows the charging and discharging time of this capacitor, which takes 20 min to charge to 3 V and the same time for discharging, thereby indicating a stable energy harvesting operation. At different current densities, the capacitor takes a different time to fully discharge, which is shown in Fig. 11j. The overall efficiency and energy storage efficiency of the designed capacitor are presented in Fig. 11k, which indicates that the resulted efficiencies are stable over the cycles. The photovoltaic response of the fabricated device provides a high electrical potential of 3.95 V, along with a photoconversion efficiency of 10.2% that leads to the dual functioning of light-harvesting and energy storage. A comparison of this device with different integrated systems based on an overall efficiency and voltage is represented in Fig. 11l, which indicates the superior efficiency and power delivering capability of the device. The fabricated wearable strain sensor can offer accurate and uninterrupted data delivery from physiological signals without any extra power sources, which leads interest in its use for developing smart sensor systems. Another flexible strain sensor has been developed by integrating a graphene/eco flex film with a meandering zinc wire that can provide an electrical potential and a current of 0.83 V and 75 µA, respectively [137]. The responsivity of the sensor is rapid without any need for an external power source, which can sense motion of a knee joint.

**Fig. 12:**
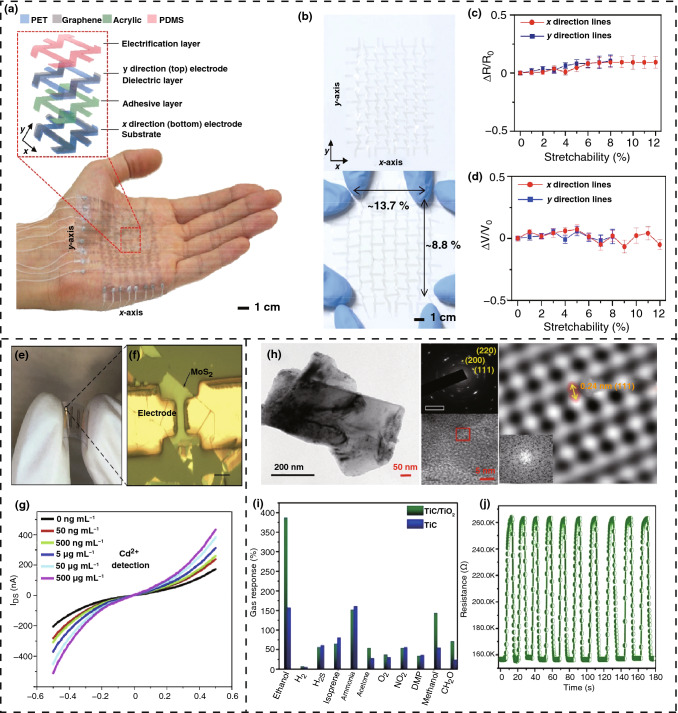
Nanogenerator-based sensors based on 2D nanomaterials. **a** Microscopic diagram of the fabricated device along with graphene electrodes. **b** Stretching behavior and extension lengths in both directions of the triboelectric nanogenerator sensor. **c** Relation between stretch level and resistance. **d** Variation of output voltage with applied stretch. **e** Chip photograph. **f** Microscopic image of the MoS_2_ sensor. **g** Effect of concentration of Cd^2+^ ions on sensor response. **h** Transmission electron microscopic photo of the selected area electron diffraction patterns and the orientation of planes. **i** Selectivity of the device. **j** Reproducibility of the fabricated sensor in ethanol exposure up to 10 cycles. Figures reprinted with permission from: **a**, **b**, **c**, **d** Ref. [137], © 2019 Elsevier; **e**, **f**, **g** Ref. [138], © 2019 Elsevier; **h**, **i**, **j** Ref. [139], © 2019 American Chemical Society

A graphene-based stretchable/wearable touch sensor powered by an inbuilt triboelectric nanogenerator (TENG) was investigated for perceiving its correlation with human skin motion, as illustrated in Fig. 12a. The bidirectional upper and lower electrodes with a dielectric layer in the auxetic form are located between them [138]. Figure 12b displays the physical shape of the device and after applying a tensile strain along x- and y-direction of the device at 13.7% and 8.8%, respectively, under a stretching interval of 1%. The resulting related resistance, along with the voltage generated, from the device with changing stretchability, is shown in Figs. 12c, d, respectively. By analyzing both parameters, it is clear that the fabricated TENG exhibits promising stability, with only a 1% deviation. This touch sensor can realize a simple touch, along with the velocity of touch sliding, and can show any character by using an actual trajectory mode in real time that can lead to developing smart input devices for communication systems.

A flexible MoS_2_-incorporated chemical sensor array that uses the functionalization of an ionophore has been demonstrated, which enables 29% elevated efficiency for label-free ion sensing [139]. Figure 12e shows a photograph of the chip, and Fig. 12f is an optical micrograph of the designed sensor on the PET substrate. The sensing performance, according to the changing Cd^+^ ion concentration, is illustrated in Fig. 12g, which indicates that an increased current is generated due to the increased concentration as it changes the resistivity of the MoS_2_ channel. The fabricated sensor device can detect Hg^2+^ and Na^+^ in samples, such as human sweat, enabling the opportunity for health or environmental monitoring sensor systems that can operate without any external power supply.

A new technique is implemented for fabricating a flexible and wearable ethanol gas sensor fabricated with titanium oxide (TiO_2_) grafted on 2D titanium carbide (TiC) nanosheets [140]. With a small noise-to-signal ratio, this device enables extended selectivity and also has the opportunity of tuning the sensitivity and selectivity within an extremely short period of response time. Figure 12h shows a TEM image of titanium carbide nanosheets and associated SAED (selected area electron diffraction) patterns. The designed sensor is more sensitive and selective for ethanol gas compared with other gases, as illustrated in Fig. 12i. Figure 12j represents the reproducibility and response time over 10 cycles of the fabricated sensor. The fabricated device can be used as a low-cost strip sensor. A further improvement can lead to playing a major role in printed electronic and biomedical applications for developing smart sensing technologies. A flexible pressure sensor has been developed using piezoresistive graphene/P(VDF-TrFE) heterostructure combining with an extreme responsivity of graphene and a superior deformity of the PDMS substrate, which exhibits excellent performance [141]. An additional pressure sensor has also been fabricated with an MXene–textile network structure that extends the sensitivity up to 12.095 kPa^−1^ at 29–40 kPa and 3.844 kPa^−1^, which is at a minimum 29 kPa, providing an excellent responsivity of 26 ms along with improved stability of 5600 cycles [142].

## Conclusion and Future Aspects

From the initial discovery of 2D nanomaterials, researchers are continuing to deepen their understanding of these fascinating materials, improve their fabrication and integration into device architectures, and seeking new applications in our daily life from home to industry and from human health to electronic devices. The electrical, chemical, optical, and mechanical properties, of these intriguing materials, are capable of further modification using a variety of approaches such as vacancy engineering, functionalization, surface defect engineering, doping with anions or cations, and hybridization for tunable capabilities of 2D nanomaterials. As a result, 2D nanomaterials have become an optimistic option in fabricating wearable, flexible devices of small size for energy scavenging, and sensing applications where a small amount of power is sufficient for analyzing and processing signals. While there are specific limitations, such as low power density and output, synthesis challenges, and high fabrication costs, 2D nanomaterials can play a significant role in future research to meet the future energy demand, if their synthesis and properties are optimized effectively.

**Fig. 13:**
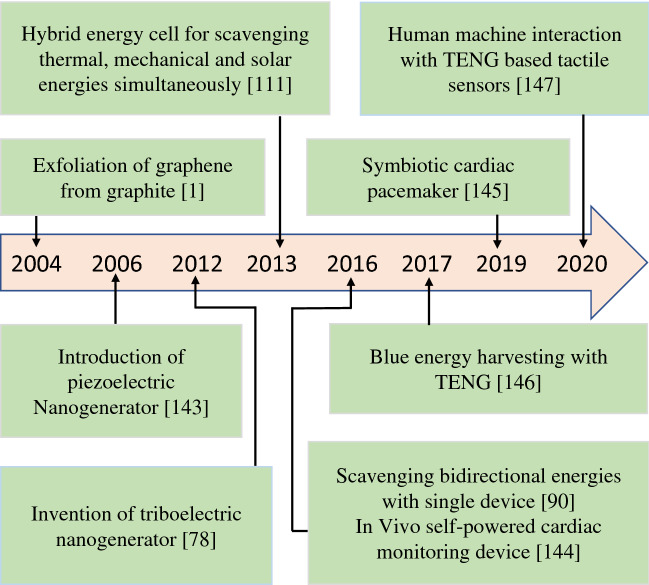
A brief timeline profile of major achievements in the field of nanogenerator research

Energy scavenging through the formation of 2D nanomaterial-based nanogenerator devices is a new technology, and the development of this field is rapid compared to other research areas. The future of this field ensure that nanoenergy and nanosystems will become an important research field, aiming at the synthesis of new materials for effective device output and device stability. This field is of interest for applications related to electronics to medical technology from simple chips to an industry-level application for various self-powered sensors. The recent and major achievement that leads nanogenerator is shown in Fig. 13. While the concept of piezoelectricity is well known, the first piezoelectric nanogenerator was proposed in 2006 [143], but within a short time frame, it was successful to draw the attention of the scientific community globally. More research has been carried away to design flexible nanogenerators based on piezoelectric, triboelectric, thermoelectric, and pyroelectric effects for wearable and portable electronics for replacing conventional batteries. After the invention of the triboelectric nanogenerator (TENG) in 2012, there has been significant interest in using the approach for scavenging mechanical energies. The hybridization of mechanical, thermal, and solar energy [111] in 2013, bidirectional energy harvesting TENG, and in vivo cardiac monitoring device based on TENG [144] in 2016 opens up diverse application areas for the technology. The blue energy harvesting concept by TENG is another major milestone in 2017 for solving the energy crisis from the sea waves [146]. Most recently, a triboelectric nanogenerator based on an implantable symbiotic heart pacemaker device was fabricated in 2019, which demonstrated the capability of nanogenerators in medical sectors [145]. In 2020, a human–machine interfacing tactile sensor has been fabricated based on a triboelectric nanogenerator, which can be an excellent opportunity to develop artificial intelligence to upgrade it to the next level as nanogenerator makes the power supply network simpler to reduce the complication of the sensor devices implanted in robots [147]. Therefore, nanogenerator-based scavenging devices have attracted siginficant attention and for the fabrication of nanodevices, and the importance of 2D nanomaterials will continue to increase in the coming future.

In this review, several types of 2D nanomaterials with unique properties have been described with the basic structure and approaches to improving the required properties by different techniques for energy scavenging. A wide range of possible methods of energy scavenging are possible using these materials. This includes solar, mechanical, thermal, and chemical energy using 2D nanomaterial-based PV cells, perovskite solar cells, water-splitting, triboelectric nanogenerators (TENG), piezoelectric nanogenerators (PENG), thermoelectric generators, pyroelectric generators, and osmotic power generation. These approaches have been explained, along with their basic mechanism and device descriptions. In addition to performance analysis, relevant modifications and extension of the devices are also discussed for achieving extended efficiency in energy scavenging. Finally, practical 2D nanomaterial-based energy scavenging devices for sensor design, nanosystems, and medical sciences are discussed. The major advantages of these devices are the opportunity to make them self-powered, wireless signal providers, miniature in size, and compatible with the human body during their operation. In addition, hybridization of these devices can be used for multi-functional applications by reducing the complexity in nanosystems and providing networks with higher flexibility in operation.

In summary, the energy scavenging techniques based on 2D nanomaterials utilizing sustainable sources are continuing to develop. A number of new materials, after the discovery of graphene, are being synthesized and studied. An significant number of investigations are being carried out by scientists to introduce highly efficient harvesting mechanisms and fabrication processes of energy scavenging devices. The performance of devices continues to improve with new and more favorable materials with fascinating properties and structures. However, further studies and investigations are needed on (i) the improvement of the performance and stable operation of these devices; (ii) the synthesis and fabrication techniques need to be more developed for practical applications; (iii) lowering the synthesizing and fabrication cost; (iv) hybridizing more scavenging techniques with a single device; (v) further investigation on new 2D nanomaterials as they develop. There is potential for energy scavenging devices based on 2D to be a major portion of self-powered sensors, nanosystems, and human–machine interaction applications. The dream of producing larger-scale energy will be also possible with more dedicated research and technological advancement in the future, which will meet the world energy demand, while also powering low power electronics without harming the ecological balance and human health.
